# The three-stage rock failure dynamics of the Drus (Mont Blanc massif, France) since the June 2005 large event

**DOI:** 10.1038/s41598-020-74162-1

**Published:** 2020-10-15

**Authors:** Antoine Guerin, Ludovic Ravanel, Battista Matasci, Michel Jaboyedoff, Philip Deline

**Affiliations:** 1 Risk Analysis Group, Institute of Earth Sciences, University of Lausanne, 1015 Lausanne, Switzerland; 2 EDYTEM Laboratory, CNRS, University Savoie Mont-Blanc (USMB), 73370 Le Bourget du Lac, France

**Keywords:** Natural hazards, Geomorphology

## Abstract

Since the end of the Little Ice Age, the west face of the Drus (Mont Blanc massif, France) has been affected by a retrogressive erosion dynamic marked by large rockfall events. From the 1950s onwards, the rock failure frequency gradually increased until the large rockfall event (292,680 m^3^) of June 2005, which made the Bonatti Pillar disappear. Aiming to characterize the rock failure activity following this major event, which may be related to permafrost warming, the granitic rock face was scanned each autumn between October 2005 and September 2016 using medium- and long-range terrestrial laser scanners. All the point clouds were successively compared to establish a rockfall source inventory and determine a volume-frequency relationship. Eleven years of monitoring revealed a phase of rock failure activity decay until September 2008, a destabilization phase between September 2008 and November 2011, and a new phase of rock failure activity decay from November 2011 to September 2016. The destabilization phase was marked by three major rockfall events covering a total volume of 61,494 m^3^, resulting in the progressive collapse of a new pillar located in the northern part of the June 2005 rockfall scar. In the same way as for the Bonatti Pillar, rock failure instability propagated upward with increasing volumes. In addition to these major events, 304 rockfall sources ranging from 0.002 to 476 m^3^ were detected between 2005 and 2016. The temporal evolution of rock failure activity reveals that after a major event, the number of rockfall sources and the eroded volume both follow a rapid decrease. The rock failure activity is characterized by an exponential decay during the period following the major event and by a power-law decay for the eroded volume. The power law describing the distribution of the source volumes detected between 2005 and 2016 indicates an exponent of 0.48 and an average rock failure activity larger of more than six events larger than 1 m^3^ per year. Over the 1905–2016 period, a total of 426,611 m^3^ of rock collapsed from the Drus west face, indicating a very high rock wall retreat rate of 14.4 mm year^−1^ over a surface of 266,700 m^2^. Averaged over a time window of 1000 years, the long-term retreat rate derived from the frequency density integration of rock failure volumes is 2.9 mm year^−1^. Despite difficulty in accessing and monitoring the site, our study demonstrates that long-term surveys of high-elevation rock faces are possible and provide valuable information that helps improve our understanding of landscape evolution in mountainous settings subject to permafrost warming.

## Introduction

Over the past two decades, many rock avalanches and large rockfall events have affected permafrost within mountain ranges around the world^1–6^, including the Alps^7–13^, as illustrated by the devastating 3.1 Mm^3^ event^14–16^ in Piz Cengalo (Switzerland) in August 2017. In high mountain areas, permafrost and its degradation (warming) due to climate change are increasingly perceived as having a fundamental role in rock wall destabilization^17–19^. The increase in the frequency and volume of rockfalls and rock avalanches—presumably validating this role—is largely suggested by the multitude of recent major events. The rockfall frequency has been verified in the Alps^20–22^, while that of volume has started to be validated in Alaska^23^.

Despite these efforts, the volume-frequency relationship in high mountain areas remains little studied because of the lack of systematic data on rockfalls^24^. Only recent research conducted in the Mont Blanc massif has made it possible to propose initial conclusions based on a large data set on rockfalls documented by a network of observers^25,26^ or by using their correlative deposits on glacial surfaces identified by satellite imagery^27^. These inventories, especially those^28^ associated with the heat wave years 2003 and 2015, showed that rockfalls were numerous but involved limited volumes (160 rockfalls > 100 m^3^) and occurred within permafrost-affected areas. Although nonexhaustive, the data collected in these databases suggest a sudden and remarkable deepening of the active layer (top layer that thaws during the summer season) together with hydrostatic pressure related to thaw, extreme rain or ice expansion before melting^29^ and advective heat transport by water percolation along discontinuities at depth.

More generally, historical rockfall inventories show that the cumulative distribution of volumes mainly follows power-law relationships^24,30–34^, except for volumes < 100 m^3^ and > 10,000 m^3^, which are underrepresented in the databases due to many small rockfalls not being reported^35^ and observation periods not being long enough to record large volumes^24^. Many authors^36–40^ who have established a rockfall inventory using remote sensing techniques, such as terrestrial laser scanning (TLS) or structure-from-motion (SfM) photogrammetry, have also put these power-law relationships forward. Nevertheless, two recent studies^41,42^ demonstrated that the monitoring interval played a key role in the completeness and correctness of rockfall inventories derived from remote sensing surveys. Thus, within the framework of an infrequent monitoring interval (typically one year), the number of detected rockfalls and their individual volumes can both be distorted by the effects of coalescence and superimposition of events^42^. However, it should be specified that the two abovementioned effects do not affect the values of cumulative eroded volumes and resulting cliff retreat rates. To use adequate terminology, it is therefore necessary to dissociate the terms *rockfall* (which relates to the fall itself^43^), *rockfall event* (referring to a specific event to which an identified source corresponds), *rockfall source* (which corresponds to a detected event that may be affected by the effects of coalescence and superimposition), and *rockfall scar* (which represents the detachment surface of one or more rockfall sources and whose volume estimates are listed in inventories).

Despite the many case studies mentioned at the beginning of the introduction, the evolution of a high-elevated rock wall following a significant destabilization has not yet been the subject of specific research. However, surveying a large rockfall scar is very helpful to evaluate the frequency and volume of mechanical readjustments within and around the scar since the change in stress fields could have consequences beyond the scar itself as suggested, for instance, the effects of glacial retreat on rock slopes (glacial debutressing, i.e.*,* lateral stress release resulting from ice melting^44–46^). Furthermore, in the context of permafrost, the possible development of a new active layer after the collapse of a significant thickness (> 10 m) can be assessed by quantifying the morphological evolution of the large scars.

To address this need, we monitored the west face of the Drus (3754 m a.s.l.), an iconic peak of the Chamonix-Mont-Blanc Valley (Mont Blanc massif, France), using a medium-range TLS and then a long-range TLS. The Drus west face is subvertical, 1000 m high and consists of Hercynian granitic rocks. It was affected by several significant collapses during the second half of the twentieth century and by a large rockfall event^20,47^ (292,680 m^3^) on 29–30 June 2005, which completely wiped out the Bonatti Pillar and significantly modified the morphology of the rock face. Rock failure activity that affected the west face of the Drus following this major event is analyzed in detail using the diachronic comparison of 12 high-resolution 3D models, which were acquired yearly between October 2005 and September 2016.

## Study site

### Geological setting

**Figure 1:**
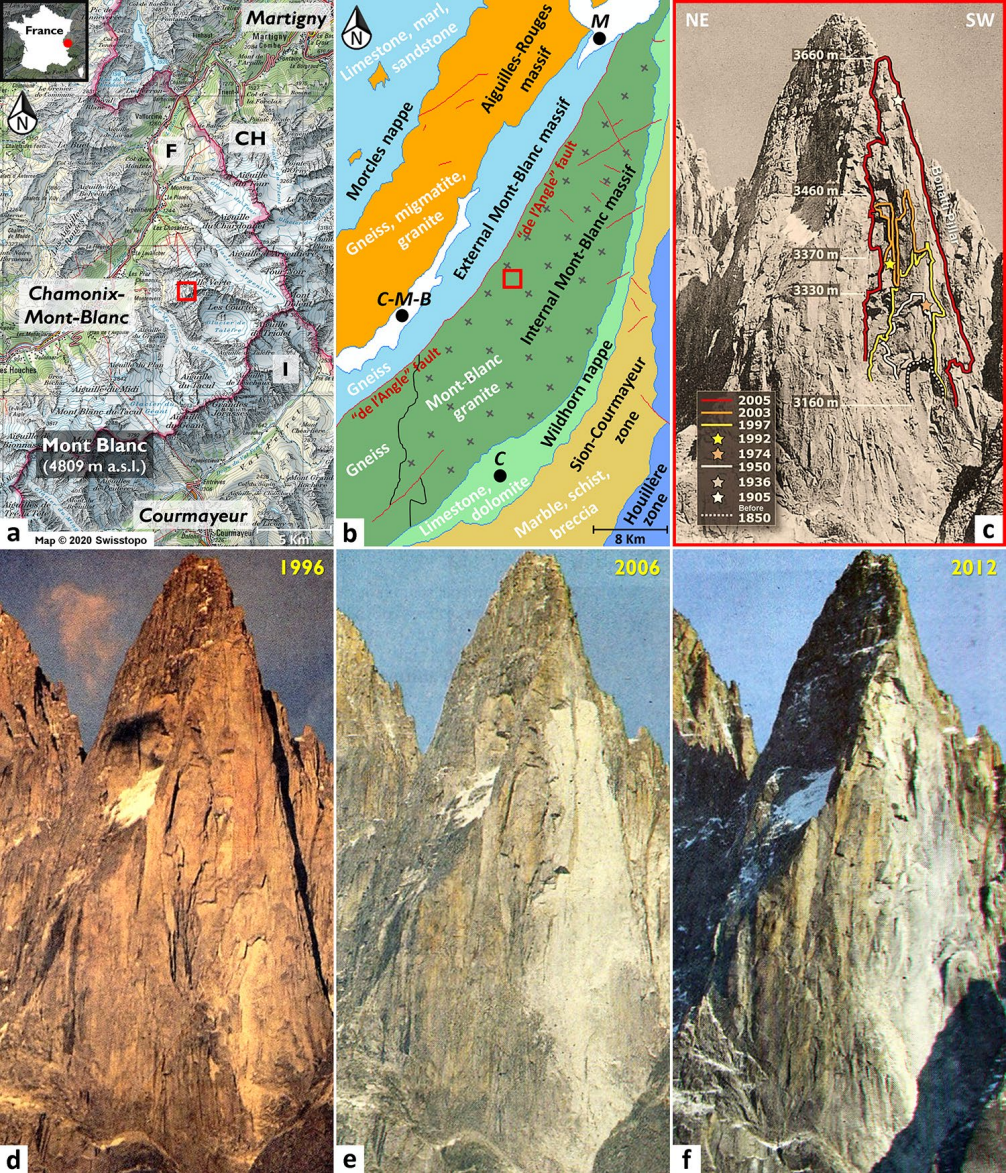
Location, geologic setting and rockfall event history of the study area. (**a**) Topographic map of the Mont Blanc massif located in the northwestern Alps between France (F), Italy (I), and Switzerland (CH); topographic credit: 2020 Swisstopo. The red frame indicates the location of the Drus (3754 m a.s.l.) within the Chamonix-Mont-Blanc Valley. (**b**) Geotectonic map of the Mont Blanc massif. This map was produced using vector data from the Swiss western Alps’ tectonic map^119^ at 1:100,000. C = Courmayeur; C-M-B = Chamonix-Mont-Blanc; M = Martigny. (**c**) Spatiotemporal reconstitution (elevation in m a.s.l.) of the main historical rockfall events that occurred in the west face of the Drus between 1850 and 2005. Estimated volumes^20,47^: 1905 = 9000 m^3^; 1936 = 5500 m^3^; 1950 = 20,000 m^3^; 1974 = 350 m^3^; 1992 = 1750 m^3^; 1997 = 27,500 m^3^; 2003 = 6500 m^3^; 2005 = 292,680 m^3^. (**d**–**f**) Evolution of the face between 1996 and 2012; photographic credit: Éric Vola (photographs reproduced under an open access license CC BY). Three major collapses affected it in Jun. 2005, Sep. 2011 and Oct. 2011.

The Mont Blanc massif is a mountain range characterized by an extraordinary combination of glaciers, rock walls and peaks, of which a dozen exceed 4000 m a.s.l. From a geographical point of view, the Mont Blanc massif (550 km^2^) is located in the northwestern Alps between France, Italy, and Switzerland (Fig. 1a). The Drus (3754 m a.s.l.) are located northeast of the town of Chamonix (Haute-Savoie, France) and consist exclusively of the Mont Blanc granite belonging to the Helvetic crystalline basement of the internal Mont Blanc massif^48,49^ (Fig. 1b). The Mont Blanc granite outcrops into a large lenticular structure that extends along a northeast-southwest axis over 37 km, which is delimited to the NW by the *faille de l’Angle*^50^ (“de l’Angle fault” in Fig. 1b) and to the SE by the para-autochthonous sedimentary cover of the Wildhorn nappe (Fig. 1b). The Mont Blanc granite is a coarse-grained calc-alkaline granite dating back to 305 ± 2 million years^51–53^, which formed during the Hercynian orogeny. In the west face of the Drus, the Mont Blanc granite presents a very fractured facies (Fig. 1c–f) mainly characterized by two very persistent subvertical joint sets (mean trace length of 80 m) oriented 238°/68° and 303°/79°, respectively^20,47,54^. In combination with many deep overhangs (and especially those oriented 106°/33°), the morphology of the west face is carved by dihedral structures that promote the destabilization of large rock compartments.

### Morphological evolution of the west face between 1850 and 2005

**Figure 2:**
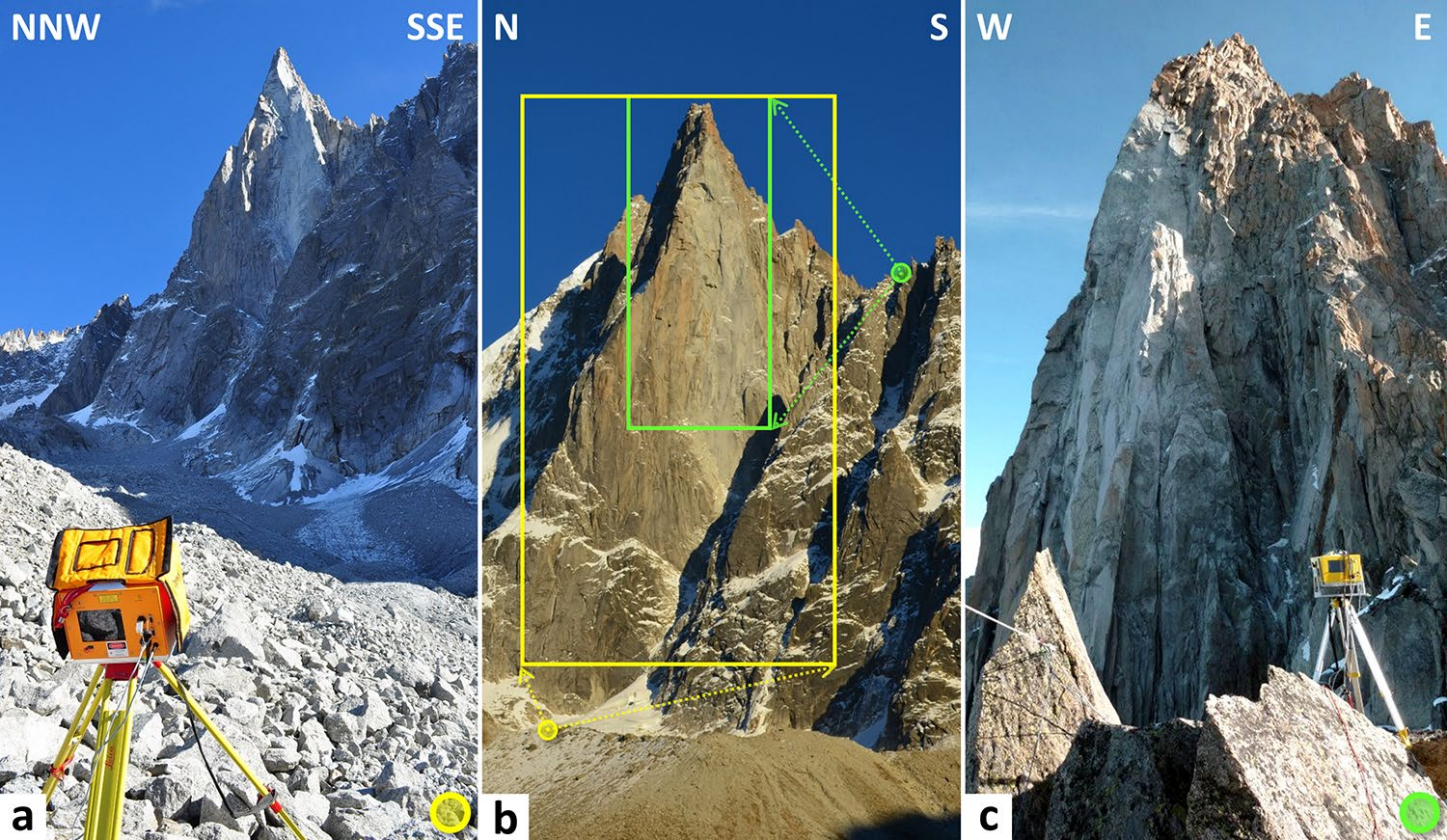
Terrestrial LiDAR data collection performed between Oct. 2005 and Sep. 2016. (**a**) Viewpoint used from Nov. 2011 to Sep. 2016. The Optech ILRIS-LR scanner is positioned on the northwestern lateral moraine of the Drus glacier (DG; location in Panel (**b**) at 2520 m a.s.l. From this viewpoint, the average distance with the rock wall is 1 km. (**b**) Location of both viewpoints (circles) and limits of the corresponding scanned surfaces (frames). Green color is attributed to the 2005–2011 period, and yellow color is attributed to the 2011–2016 period; the scanned rock surfaces are 108,400 m^2^ and 266,700 m^2^, respectively. (**c**) Viewpoint used from Oct. 2005 to Sep. 2011. The Optech ILRIS-3D scanner is positioned on the Flammes de Pierre ridge (FP; location in Panel (**b**)) at 3360 m a.s.l. From this viewpoint, the average distance from the rock wall is 400 m.

The first photographs of the west face of the Drus (in the form of glass plates essentially) were taken^20^ from 1850 to 1870. These historical documents determined that the first rockfall scar (located approximately 3160 m a.s.l.; Fig. 1c) that affected the Bonatti Pillar was prior to 1850. From the end of the Little Ice Age to the middle of the twentieth century, only three major rockfall events were identified in 1905, 1936 and 1950 (Fig. 1c). The volumes estimated^20^ are 9000 m^3^, 5500 m^3^ and 20,000 m^3^, respectively. In addition, it is worth noting that the 1934 and 1950 rockfall scars are located directly below and above the limits of the 1850 rockfall scar (Fig. 1c). From the 1950s onwards, the rock failure dynamics progressively accelerated as five major events were detected between 1974 and 2005, including four between 1992 and 2005 (Fig. 1c). Typical of progressive overhang-type failures (e.g., the 1920 rockfall events that occurred on the Italian side of the Mont Blanc massif in the east face of the Grand Pilier d'Angle^12,55,56^), the rockfall events propagated upward from the location of the 1950 scar. Despite a slight decline in 2003, the volumes associated with the 1974–2005 period gradually increased (Fig. 1c), resulting in the complete disappearance of the Bonatti Pillar on 29–30 June 2005 due to a large rockfall event^20,47^ of 292,680 m^3^. This event generated a large 700-m-high and 80-m-wide gray scar (Fig. 1e), whose debris covered the upper part of the Drus glacier and its morainic complex (Fig. 2a), located at the base of the rock wall.

## Materials and methods

### TLS data acquisition

Thirteen TLS measurement campaigns were conducted (each autumn) between October 2005 and September 2016 with two data acquisitions in 2011. The TLS measurements from October 2005 to September 2011 (7 acquisitions) were carried out from the Flammes de Pierre ridge (FP—3360 m a.s.l.; Fig. 2b,c) using a medium-range laser scanner (Optech ILRIS-3D; specifications listed in Table 1). However, due to a technical problem (defective scanner), the September 2009 data could not be used for the diachronic comparisons. From this first viewpoint located at an average distance of 400 m from the rock wall, the collection of three scenes (acquired from the same TLS position) with an average vertical overlap of 30% was needed to scan the entire June 2005 rockfall scar (see Supplementary Fig. 1a). Due to the difficulty of access (helicopter toe in drop-off) and the narrowness of the Flammes de Pierre ridge, no other TLS position could be used to complete the point clouds (i.e., fill in holes due to areas masked by the relief). From November 2011, all TLS measurements (6 acquisitions) were acquired from the northwestern lateral moraine of the Drus glacier (DG— 2520 m a.s.l.; Fig. 2a,b) using a long-range laser scanner (Optech ILRIS-LR; specifications listed in Table 1). This second viewpoint was located at an average distance of 1 km from the rock wall, and a single scene was sufficient to scan the entire west face. The two laser scanners have a manufactured-specified accuracy^57^ of 7 mm for the distance to a single point at a range of 100 m (Table 1). In terms of resolution, the resulting TLS data consist of an average of 10.1 million points over the 2005–2011 period and an average of 17.3 million points over the 2011–2016 period. When compared to the respective scanned surface areas (108,400 m^2^ then 266,700 m^2^), these values give an average surface density of 93 pts/m^2^ (i.e., a point-to-point spacing of 10.4 cm; see Supplementary Fig. 1b) for point clouds acquired from the FP viewpoint and of 58 pts/m^2^ (i.e., a point-to-point spacing of 13.1 cm; see Supplementary Fig. 2a) for point clouds acquired from the DG viewpoint. However, in the overlapping areas (case of the scenes acquired from the FP viewpoint) and in the areas closest to the TLS position (e.g., the lower part of the west face for the DG viewpoint), the surface density is locally higher. Thus, within these areas, the surface density can reach over 150 pts/m^2^ for the FP viewpoint (i.e., a point-to-point spacing of 8.2 cm; see Supplementary Fig. 1b), and over 90 pts/m^2^ for the DG viewpoint (i.e., a point-to-point spacing of 10.5 cm; see Supplementary Fig. 2a).

**Table 1:** Main specifications of the two terrestrial laser scanners used.

Parameter	Optech ILRIS-3D	Optech ILRIS-LR
Range (80% reflectivity)	1200 m	3000 m
Range (10% reflectivity)	400 m	1330 m
Laser repetition rate	2500 Hz	10,000 Hz
Raw range accuracy	7 mm @ 100 m	
Raw angle accuracy	8 mm @ 100 m (80 µrad)	
Field of view	40° × 40°	
Maximum density (point-to-point spacing)	2 cm @ 1000 m	
Beam diameter	22 mm @ 100 m	27 mm @ 100 m
Beam divergence	0.009740° (170 µrad)	0.014324° (250 µrad)
Laser wavelength	1535 nm	1064 nm

### TLS data registration

Due to the absence of vegetation at that altitude, data registration required four steps: (1) point-to-point coarse alignment on the October 2005 georeferenced point cloud^47^; (2) precise point-to-point alignment considering only the stable parts; (3) generation of the successive references meshes; and (4) point-to-surface alignment on stable parts. Step 1 consisted of manually selecting several pairs of common points (at least three) between the models at *t*_*i*_ (reference acquisition) and *t*_*i*+1_ to roughly align the point clouds. Step 2 involved a manual selection of the stable parts (areas outside the June 2005 rockfall scar, see Supplementary Fig. 2b), followed by application of the point-to-point iterative closest point (ICP) algorithms^58,59^ implemented in CloudCompare^60^ software. Point-to-point ICP algorithms aims at finding the geometric transformation between a point cloud to be align and a reference point cloud (considered fixed), by minimizing the mean square errors between the corresponding points^58,59^. The term “iterative” derives from the fact that the correspondences are reconsidered as the solution comes closer to the error local minimum. Basically, the iterative ICP algorithms’ steps are^61,62^: (a) searching for matches between the two point clouds; (b) estimating of a roto-translation matrix using a root mean square point-to-point distance metric minimization technique, which will best align each point to be align to its match found in the reference point cloud; (c) transforming the point cloud to be align using the determined geometric transformation; and (d) iterating of the three previous steps to minimize the root mean square error. Once the stable parts are aligned, all the areas considered potentially unstable were transformed according to the roto-translation matrices determined for the stable parts. Alignment accuracy being one of the leading error sources affecting change detection between two 3D models^38,63,64^, the point-to-surface iterative ICP algorithms^65^ have also been used. Thus, we transformed each reference acquisition into a triangular mesh using the Poisson surface reconstruction algorithms^66^ implemented in 3DReshaper^67^ software. This third step was carried out by keeping all the points of each TLS model (no subsampling) and choosing a maximum length of 3 m for the triangle edge to fill most existing holes in the point cloud (areas masked by the terrain relief). When applied over exactly the same stable areas defined in step 2, point-to-surface ICP algorithms optimize the registration required for point-to-mesh change detections. At the end of this process, a point-to-surface standard deviation of ± 3.5 cm (confidence interval given by 2σ) characterizes the alignment of 2005–2011 data in stable areas. Over the 2011–2016 period (DG viewpoint), the point-to-surface standard deviation is ± 4.8 cm.

### Change detection and noise filtering

Surface changes between two 3D models were determined by the calculation of the shortest orthogonal distance^68^ between a point and the nearest triangle of the mesh. Nevertheless, to smooth the residual error induced by the instrumental measurement noise and/or by poor atmospheric conditions (e.g., rising mist or hot air circulation^69^), a spatial noise filter^70^ was applied to the raw distances. Based on the nearest neighbor averaging method^71,72^, this algorithm allows denoising of the raw distance values by filtering the instrumental error^70^. In this study, the averaging process was performed using the nearest 100 neighbors. After noise filtering, the point-to-surface standard deviations (2σ) in stable areas were ± 2.7 cm for the 2005–2011 period and ± 3.5 cm for the 2011–2016 period. Although a last step of noise filtering is applied during the rockfall source extraction (see step 3 described in the following section), we used the abovementioned values to define the level of detection at 95% (LoD_95%_) of the filtered point-to-mesh comparisons.

### Rockfall source identification and volume calculation

Extraction of the two point clusters belonging to rockfall sources (collapsed surface and scar; see Supplementary Fig. 3a) was performed using a semi-automatic method^47^. The first two steps of this method are relative to the LoD_95%_ defined above and include (1) attribution of three colors to separate the negative and positive deviations located on both sides of the LoD_95%_ into three categories (which alone represents one category), and (2) splitting of the 3D model into three distinct parts to keep only the point clusters with a negative difference greater than the LoD_95%_. The last two steps are common to another method^73^ recently developed and use the following two algorithms: (3) the Nearest-Neighbor Clutter Removal algorithm^74^ is used to separate the residual noise and the points belonging to rockfall sources into two classes, and (4) the density-based clustering algorithm^75^ DBSCAN is used to individualize each point cluster of the “rockfall sources class”.

All rockfall source volumes were calculated by generating closed triangular meshes using 3DReshaper software. The volume is calculated as the sum of all the tetrahedron volumes contained inside the closed mesh. For each volume, the following three steps were performed: (a) generation of two separate meshes (collapsed surface and scar) by keeping all the points of the two extracted clusters (see Supplementary Fig. 3b); (b) generation of a third mesh connecting the outer contours of the first two meshes and filling holes that may be present within them (see Supplementary Fig. 3c,d); and (c) merging of the three meshes obtained. Nevertheless, it should be specified that filling of the holes due to areas masked by the relief or attenuation of the laser beam with increasing range during TLS data acquisition concerned only 9% of the detected rockfall events (including the three largest). As noted by three recent studies^76–78^, hole filling, which involves 3D surface reconstruction, is a crucial step that influences volumetric calculations and hence rockfall source volume-frequency relationships. In addition, ensuring that a mesh is topologically correct (i.e., fully watertight (free of holes), free of intersecting or overlapping triangles, and with consistent normal orientation) require significant manual editing which is time-consuming^76,77^. Despite the various semi-automatic methods^76,77,79,80^ developed for this purpose in recent years, manual and individual hole filling was performed in this study. Thus, each hole has been filled with large triangles constrained by the radius of curvature fitting at best the orientation of the facets located at the hole edge (see Supplementary Fig. 3c,d). Although this approach involves a degree of subjectivity, being able to manually test several solutions of 3D surface reconstruction by holes allows to adapt to the morphology of each occlusion and to reconstruct as well as possible the topographic surfaces not scanned.

The volumetric error is defined as the sum of the errors associated with the volume of each tetrahedron, which involves the product of the area of its triangular base by its height. Thus, the uncertainty in volume estimates is influenced by the chosen LoD_95%_ (which depends on the registration error and amount of residual noise) and by the surface area (which depends on the shape) of each rockfall source. Therefore, considering the abovementioned LoD_95%_ and average point-to-point spacings, the minimum detectable volumes of rockfall source associated with each viewpoint are 2.9 × 10^−4^ m^3^ and 6.0 × 10^−4^ m^3^, respectively. However, these values are given as an indication since they correspond to collapsed surfaces delimited by an agglomeration of only four points (i.e., two triangles). For this study, we considered that at least a surface agglomeration of 6 to 8 points was needed to be certain that it was a true rockfall event. Based on this assumption, the filtered minimum detectable volumes of rockfall source are between 5.8 × 10^−4^ and 1.1 × 10^−3^ m^3^ for the FP viewpoint and between 1.2 × 10^−3^ and 2.4 × 10^−3^ m^3^ for the DG viewpoint. To better approximate the volume, four geometric forms have been integrated into error calculations: cubic, rectangular parallelepiped, triangular prism and complex (combination of at least two of the first three geometries). Overall, although the volume uncertainty depends on the resolution of the point clouds, it appears clear (see examples in Supplementary Table 1) that smaller rockfall sources have a higher volumetric error and that very elongated shapes tend to increase the margin of uncertainty. In this study, the uncertainty values vary between 0.9 and 29.3% for the 2005–2011 period (values obtained for the maximum and minimum volumes of 17,456 m^3^ and 0.002 m^3^; Table 2) and between 1.3 and 22.8% for the 2011–2016 period (values obtained for the maximum and minimum volumes of 41,810 m^3^ and 0.01 m^3^; Table 2).

**Table 2:** Rockfall source inventory for each monitored period.

Monitoring period	Data sources for detection	Viewpoint	Number of rockfall sources	Volume range (m^3^)	Cumulative volume (m^3^)
Jun. 2005	SfM^a^–TLS	–	1	292,680	292,680
Oct. 05–Oct. 06	TLS–TLS	FP	73	0.005–475.9	556.6
Oct. 06–Sep. 07	TLS–TLS	FP	46	0.003–18.8	63.4
Sep. 07–Sep. 08	TLS–TLS	FP	18	0.002–15.6	41.4
Sep. 08–Oct. 10	TLS–TLS	FP	37	0.002–2228	2643
Oct. 10–Sep. 11	TLS–TLS	FP	74	0.002–17,456	17,913
Sep. 11–Nov. 11	TLS–TLS	FP and DG	1	41,810	41,810
Nov. 11–Oct. 12	TLS–TLS	DG	35	0.02–130.2	249.4
Oct. 12–Oct. 13	TLS–TLS	DG	5	0.16–1.4	2.2
Oct. 13–Sep. 14	TLS–TLS	DG	2	0.05–0.6	0.6
Sep. 14–Nov. 15	TLS–TLS	DG	13	0.03–2.1	5.6
Nov. 15–Sep. 16	TLS–TLS	DG	3	0.01–0.4	0.5

^a^3D model from a previous study^47^.

*FP* Flammes de Pierre ridge, *DG* Drus glacier.

### Volume-frequency relationship of rockfall sources

Once the volume has been estimated, a relationship between the magnitude and the frequency of failures can be defined. Many rockfall source volume distributions obtained from historical inventories^24,30,31,33,34^, a network of observers^28^, or high-resolution TLS monitoring^36,40,42,81,82^ show that the relationship between volume and cumulative frequency can be expressed by negative power laws of the form:

1 $$F\left( {v > V} \right) = \alpha V^{ - \beta }$$

where *F*(*v* > *V*) is the cumulative number of rockfall sources per time unit larger than the volume *V*, *α* is the intercept, and *β* is the exponent^24^. An increase in the *α*-value indicates a rise in the rock failure frequency, and an increase in the *β*-value indicates a rise in the proportion of small volumes compared with larger volumes^36^.

Rockfall source volume-frequency distributions were fitted with power laws using the maximum likelihood method, as suggested by many authors^32,42,83,84^. The maximum likelihood estimate for *β* in the case of a pure power-law distribution is defined by the following equation^85^:

2 $$\beta = \frac{1}{{\left[ {\left\langle {\log \left( V \right)} \right\rangle - \log \left( {V_{0} } \right)} \right]}}$$

with a standard deviation for *β* determined by the following equation:

3 $${\upsigma }_{\beta } = \frac{\beta }{{\sqrt {N_{0} } }}$$

where *V*_*0*_ is the minimum volume used in the power law fit, < *log*(*V*) > is the average volume of the events larger than *V*_*0*_, and *N*_*0*_ is the number of events with a volume larger than *V*_*0*_. The coefficient of determination R^2^, the sum of the squared estimate of errors (SSE) and the root mean square error (RMSE) were calculated (see Supplementary Tables 1 and 2) to test whether a power law is a plausible fit based on the values determined for *α*, *β* and *V*_*0*_. More specifically, the combination of an R^2^ value close to 1 and SSE-RMSE values close to 0 indicates that the observed data fit very well with the tested distribution law.

## Results and discussion

### Morphological evolution of the rock face between 2005 and 2016

**Figure 3:**
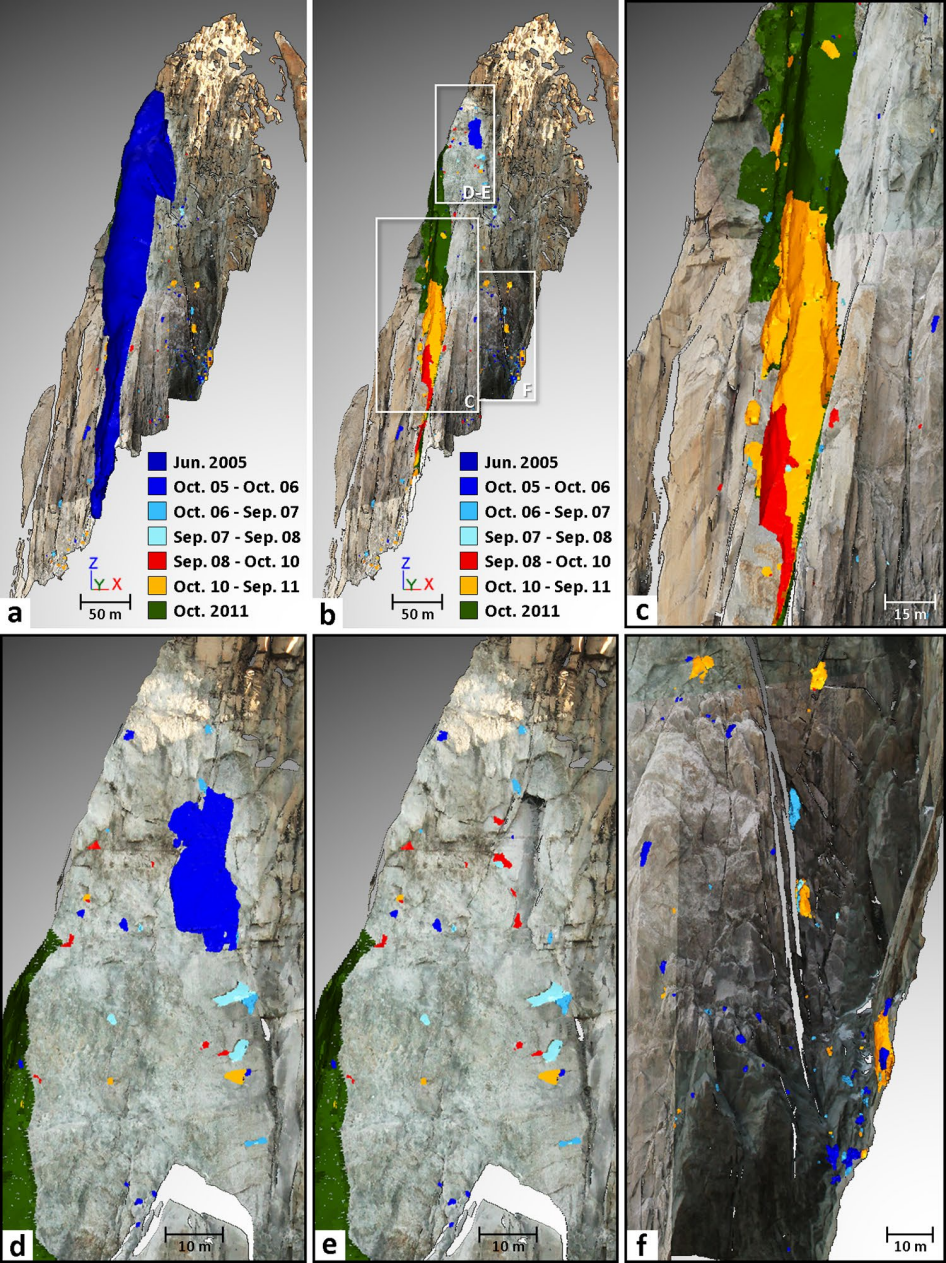
Six years of rockfall source inventory highlighted for the Drus west face by means of TLS monitoring. (**a**), (**b**) A total of 249 rockfall sources ranging from 0.002 to 41,810 m^3^ were detected between Oct. 2005 and Nov. 2011. Colors assigned to the different monitoring periods allow tracking of the spatial and temporal progression of rock failures (numbers specified in Table 2). The dark blue mesh representing the Jun. 2005 rockfall event is from a previous study^47^. Background topographic surface: Sep. 2011 mesh textured with five pictures. (**c**) Details of the collapsed pillar between Sep. 2008 and Nov. 2011. (**d**), (**e**) Details of the rock failure activity detected in the highest part of the Jun. 2005 rockfall scar. A total of 39 rockfall sources ranging from 0.002 to 476 m^3^ were detected in this area. Panel (**e**) shows the 7 rockfall sources that occurred within the rockfall scar of 476 m^3^. (**f**) Details of the impact zone located downstream of the active area shown in Panels (**d**) and (**e**). A total of 78 rockfall sources ranging from 0.005 to 67.8 m^3^ were detected in this area.

**Figure 4:**
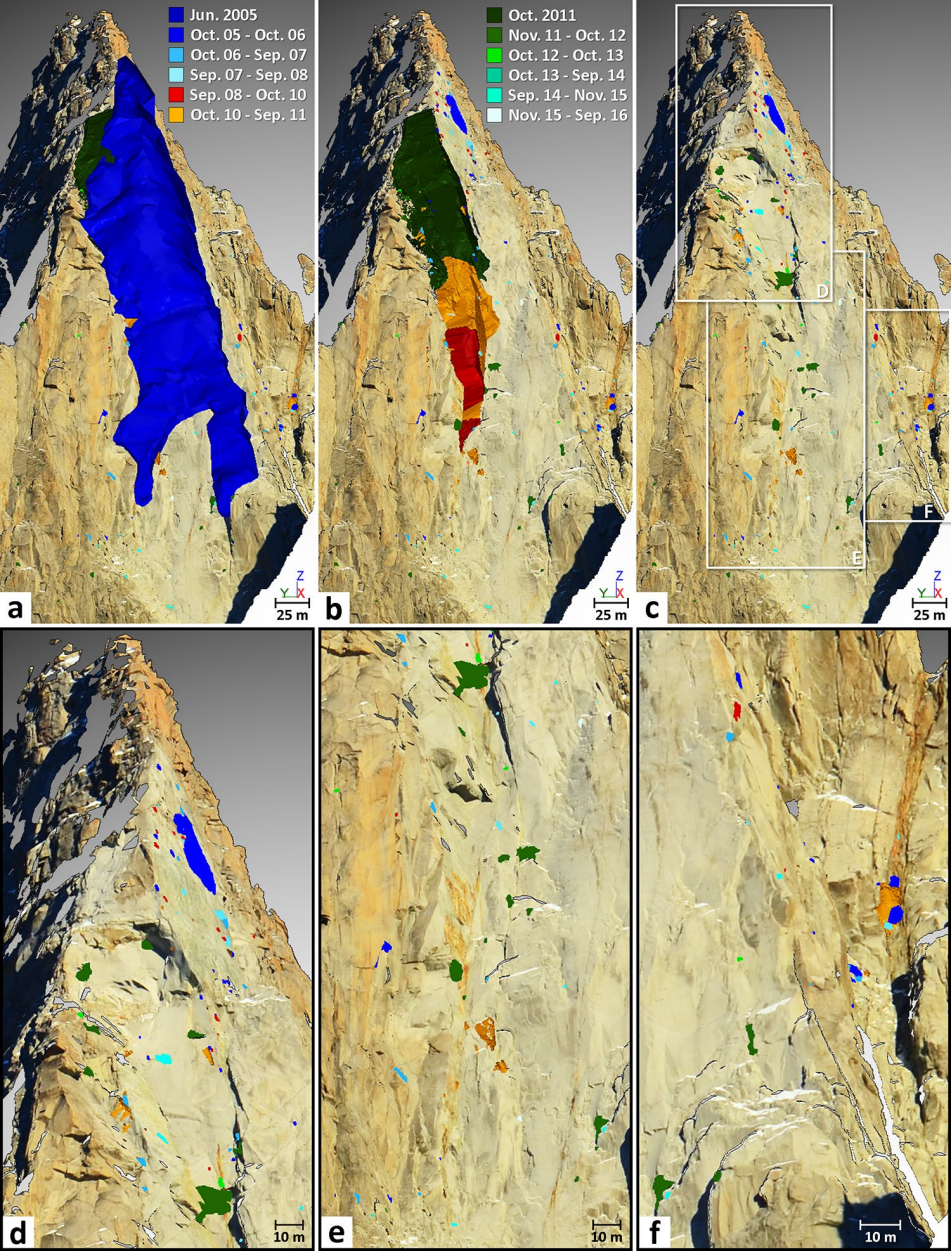
Eleven years of rockfall source inventory highlighted for the Drus west face by means of TLS monitoring. (**a**–**c**) A total of 307 rockfall sources, ranging from 0.002 to 41,810 m^3^ were detected between Oct. 2005 and Sep. 2016. Colors assigned to the different monitoring periods allow tracking of the spatial and temporal progression of rock failures (numbers specified in Table 2). The dark blue mesh representing the Jun. 2005 rockfall event is from a previous study^47^. Background topographic surface: Sep. 2016 mesh textured with a gigapixel panorama. (**d**) Details of the rock failure activity detected in the upper part of the Jun. 2005 rockfall scar. A total of 101 rockfall sources ranging from 0.002 to 41,810 m^3^ were detected in this area. (**e**) Details of the rock failure activity detected in the lower part of the Jun. 2005 rockfall scar. A total of 92 rockfall sources ranging from 0.02 to 17,456 m^3^ were detected in this area. (**f**) Details of the rock failure activity detected in the impact zone (Fig. 3f). A total of 104 rockfall sources ranging from 0.005 to 67.8 m^3^ were detected in this area.

During the 11-year investigation period, 307 rockfall sources were detected in the west face of the Drus (Table 2). Figures 3 and 4 provide an overview of the time and location of all rockfall sources detected between October 2005 and September 2016. The majority of rockfall sources (approximately three-fifths) are located within the June 2005 rockfall scar area (i.e., within 31,300 m^2^); nevertheless, there were more rockfall sources in the upper half of the scar (103 sources) than in the lower half (75 sources) (Fig. 4). The other rockfall sources are situated on both sides of the lower part of the June 2005 scar, and some of them probably correspond to the rock detached by the impact from rockfalls above (Figs. 3 and 4).

**Figure 5:**
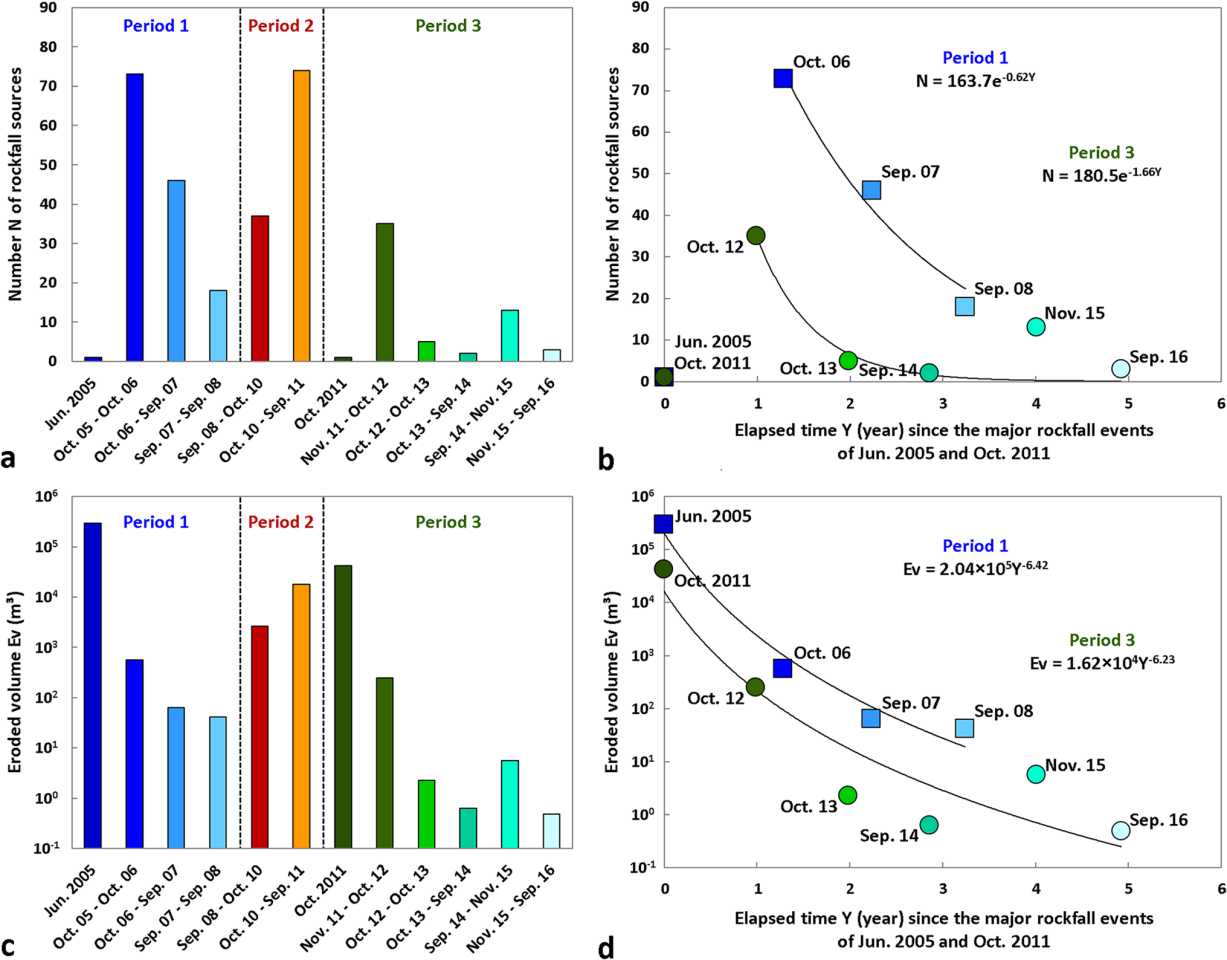
Rock failure activity statistics of the Drus west face between 2005 and 2016. Three periods have been determined: (1) the post-large rockfall event period (Jun. 2005–Sep. 2008); (2) the transition period prior to the major rockfall events of autumn 2011 (Sep. 2008–Sep. 2011; period marked by a defective scanner in Sep. 2009); and (3) the post-2011 collapse period (Oct. 2011–Sep. 2016). (**a**) Temporal evolution of the rockfall source number since Jun. 2005. (**b**) Exponential decays of rockfall source number since the major collapses in Jun. 2005 and Oct. 2011. Goodness-of-fit indicator: Period 1: R^2^ = 0.975; Period 3 R^2^ = 0.770. (**c**) Temporal evolution of the eroded volume since Jun. 2005. (**d**) Power law decays of eroded volume since the major collapses in Jun. 2005 and Oct. 2011 (semi-logarithmic graph). Goodness-of-fit indicator: Period 1: R^2^ = 0.972; Period 3: R^2^ = 0.867.

Following the large rockfall event of 29–30 June 2005 (Figs. 3a and 4a), 137 rockfall sources ranging between 0.002 and 476 m^3^ were detected between October 2005 and September 2008. Table 2 and Fig. 5a show that rock failure activity gradually decreased during this period, as the annual number of sources detected fell from 73 to 46 and then to 18. Similarly, the annual eroded volume (Fig. 5b) followed the same trend since the largest rockfall sources were detected in 2005–2006 (Table 2; Figs. 3 and 4). Thus, the period from October 2005 to September 2008 is considered to be a period of rock failure activity decay.

**Figure 6:**
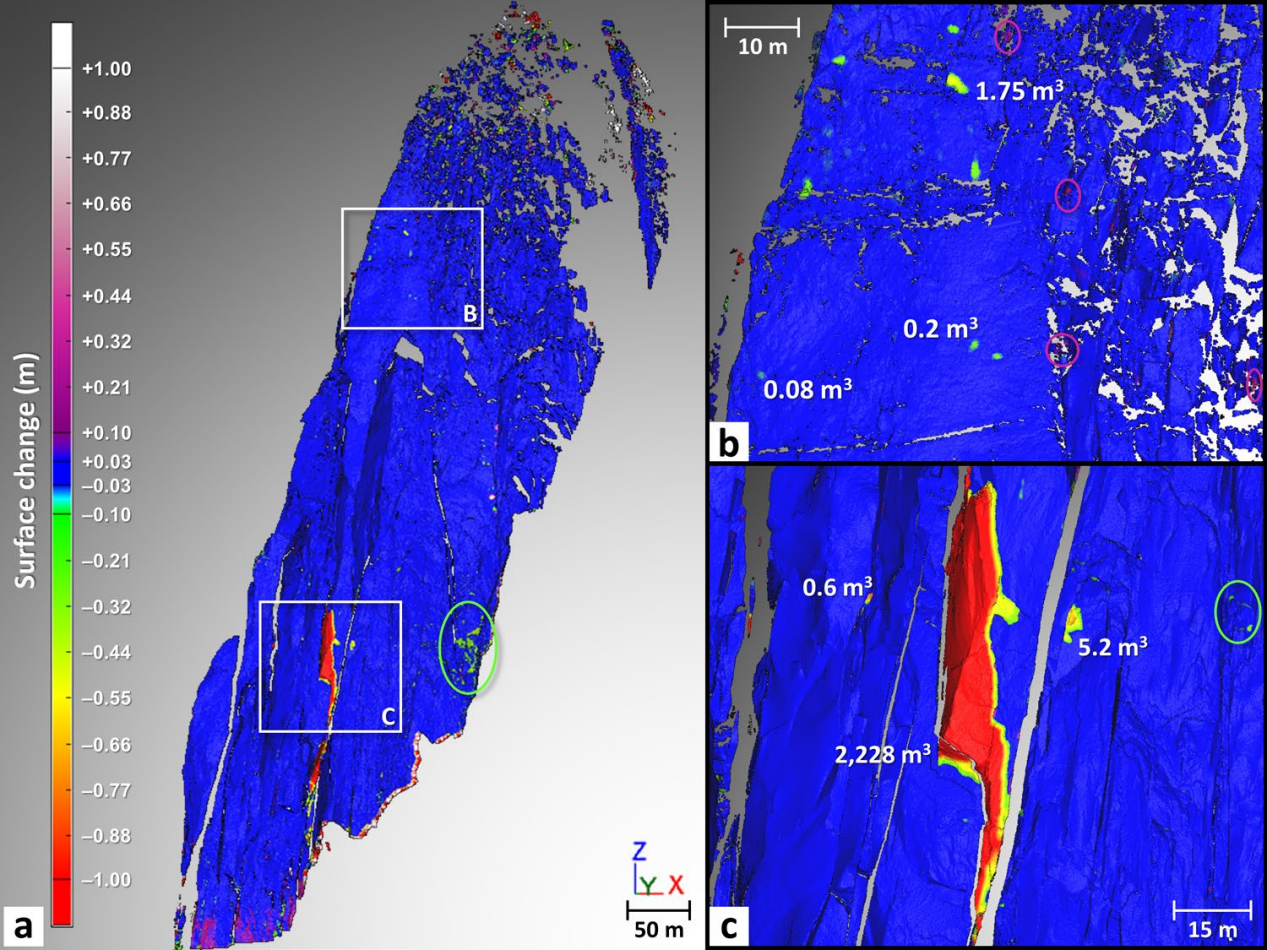
Results of the comparison between the Sep. 2008 and Oct. 2010 data. (**a**) Filtered point-to-mesh differences between the 2008 mesh and the 2010 point cloud. Positive deviations are associated with border effects (bottom of the rock face) and snow accumulation (purple ellipse-Panel (**b**)). Blue areas have differences less than the LoD_95%_ (2σ) of ± 2.7 cm. Negative surface changes correspond to either snow-melt areas (green ellipse) or to detachment areas (rockfall sources and impacts); rockfall source number and volume range are specified in Table 2. (**b**) Details of the rock failure activity detected in the highest part of the Jun. 2005 rockfall scar. (**c**) Details of the largest rockfall event (maximum thickness: 9.4 m) detected between 2008 and 2010; following this event, the rest of the pillar located above collapsed in Sep.–Oct. 2011 (Fig. 7).

**Figure 7:**
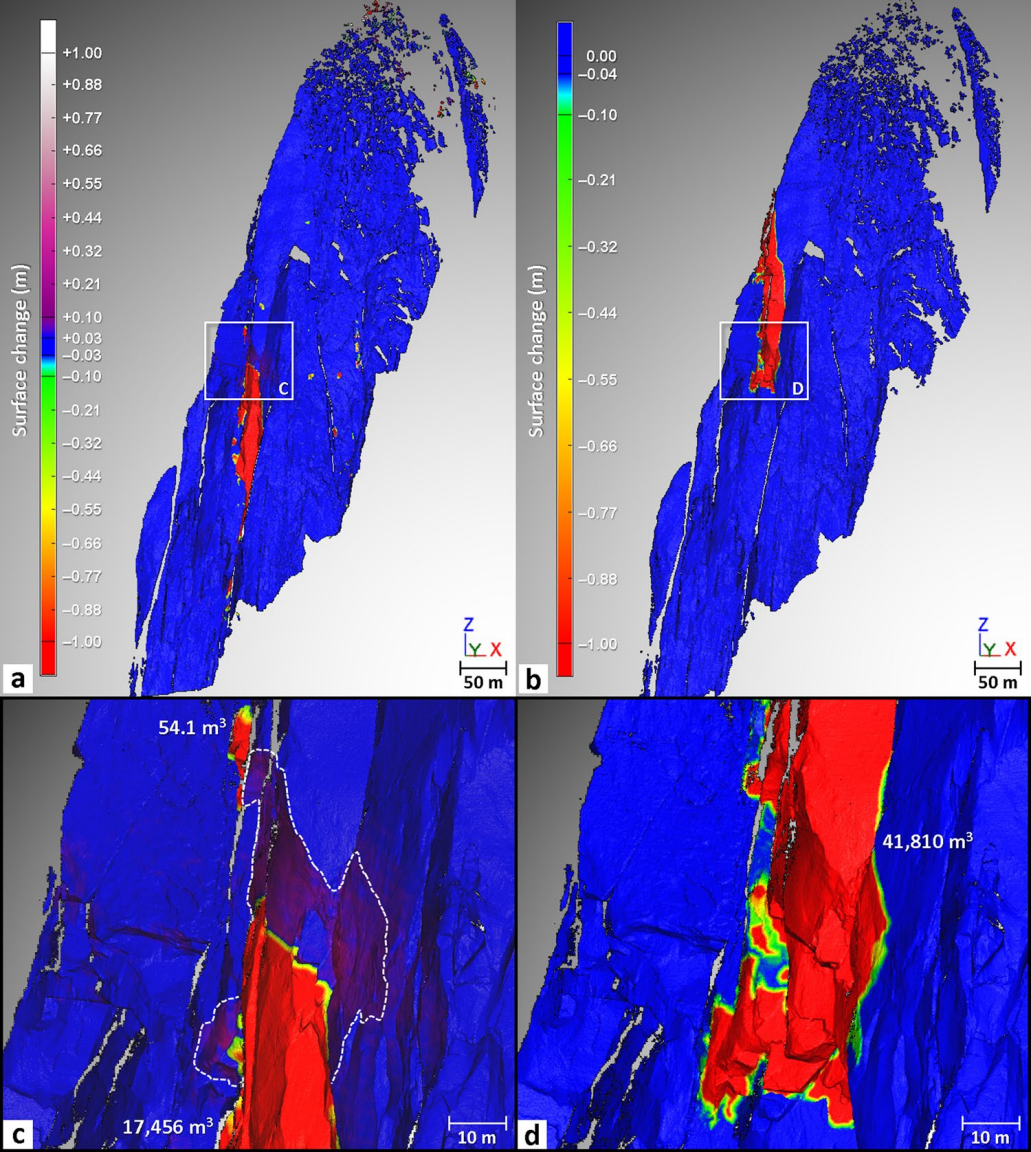
Results of the comparison between the Oct. 2010 and Nov. 2011 data. (**a**), (**b**) Filtered point-to-mesh differences between and the 2010 mesh and the Sep. 2011 point cloud and between the Sep. 2011 mesh and the Nov. 2011 point cloud (combination of both viewpoints), respectively. Positive deviations correspond to either snow accumulation areas or to areas having undergone an outward deformation (Panel (**c**)). Blue areas have differences less than the LoD_95%_ (2σ) of ± 2.7 cm and 3.5 cm (in the overlapping areas). Negative surface changes correspond to either snow-melt areas or to detachment areas; rockfall source number and volume range are specified in Table 2. (**c**) Details of the upper part of the 11 Sep. 2011 rockfall event (maximum thickness: 11.8 m). An outward deformation area (in purple) appears just above this event. The white dashed line shows the contour of the deformation area. (**d**) Details of the lower part of the 30 Oct. 2011 rockfall event (maximum thickness: 14.3 m). The lateral limits of the basal section of this event correspond closely with those of the deformation pattern highlighted in Panel (**c**).

During our monitoring, the largest rockfall events occurred between September 2008 and October 2011. Precisely dated thanks to a network of observers^26^, a first overhanging volume of 2228 m^3^, located at 3340 m a.s.l., detached on 03 September 2009 (Figs. 3, 4 and 6). Directly above this compartment, two major collapses of 17,456 m^3^ and 41,810 m^3^ occurred on 11 September 2011 and 30 October 2011, respectively (Figs. 3, 4 and 7). Although the analysis of the 2008–2010 period is biased due to the technical problem that occurred during the September 2009 acquisition and it is not known whether activity continued to decrease between September 2008 and 03 September 2009, it still appears that the rock failure activity increased between September 2008 and September 2011 (Table 2 and Fig. 5). Rising from 37 rockfall sources detected in 2008–2010 to 74 sources in 2010–2011, the rock failure activity has increased in both number and volume (Table 2 and Fig. 5) until the collapse that occurred on 30 October 2011. Consequently, the period from October 2008 to October 2011 is considered to be a destabilization phase.

The last monitoring period (November 2011-September 2016) was marked by 58 rockfall sources ranging from 0.01 to 130 m^3^ (Table 2). This period is similar to the 2005–2008 period since (1) rock failure activity (in terms of annual number and eroded volume) decreased globally from year to year (Fig. 5), and (2) the largest rockfall sources were detected during the first year following the major collapse on 30 October 2011 (Table 2). Therefore, despite the slight increase in the number and volume of rockfall sources in 2014–2015, the 2011–2016 period is also considered to be a period of rock failure activity decay.

### Analysis of the 2008–2011 rockfall event sequence

The retrogressive erosion dynamic that destroyed the 300-m-high pillar between 2008 and 2011 (Figs. 3c and 4b) is very reminiscent of the one that wiped out the Bonatti Pillar in June 2005 (Fig. 1c), for which the retrogressive erosion has likely began more than a century before, as indicated by the visible scar in photographs from the 1850s. Not only did instabilities (successive failures of overhangs) propagate upwards, but the involved volumes also increased between 2008 and 2010. A similar erosion system has recently been highlighted^40,86^ by means of TLS and SfM monitoring performed in the southeast face of El Capitan (2307 m a.s.l.) in California (Yosemite National Park, USA). This survey revealed that the major rockfall events (cumulated volume > 10,000 m^3^) of September 2017 (which left one person dead and others injured) were actually linked to a first rock failure of 650 m^3^, which occurred in October 2010 and was located in the lower part of the rock wall. Although several types of plutonic rocks (mainly granites and diorites) outcrop in the southeast face of El Capitan^87^ and rock failure activity in Yosemite National Park is often related to detachments of exfoliation sheets^35,88–91^, it is interesting to find similarities in the spatial progression of large rockfall events within granitic rock faces.

Figure 4b provides information on the amount and location of small rockfall sources that affected the 300-m-high pillar before its disappearance during autumn 2011. It turns out that 23 rockfall sources were detected within the pillar between June 2005 and September 2011 and that 90% of them were located near the pillar’s lateral limits. This kind of pre-collapse activity involving small rockfall sources near the boundaries of a future larger scar has been noticed by several authors^40–42,92–94^ and is probably induced by progressive pre-failure deformations.

The analysis of surface changes detected between September 2008 and October 2010 does not reveal any particular change pattern around the collapsed compartment of 2228 m^3^ (Fig. 6c). In other words, no short-term (time lapse smaller than the acquisition interval) precursor deformation to the 11 September 2011 rockfall event could be identified with this comparison. In addition, the only volume changes that did not correspond to rockfall sources were caused by snow accumulations (represented by positive deviations) or snow melt (negative deviations); these areas were identified by photo comparison.

The point-to-mesh comparison from October 2010 to September 2011 reveals an interesting outward deformation pattern located directly above the upper limit of the 11 September 2011 rockfall event (Fig. 7a,c). Characterized by a maximum deformation of + 9.3 cm (+ 6.5 cm on average), this deformation pattern was probably generated during or immediately after the 11 September 2011 rockfall event. Furthermore, it probably represents a precursory movement of the 30 October 2011 rockfall event since the lower limits of the deformation pattern (Fig. 7b,d) correspond very precisely to the lower limits of this event. Therefore, an outward rotational movement along the vertical axis affected the base of the remaining pillar (over the first 50 m) a month and a half before it collapsed. However, the detection of this pre-failure deformation brings to mind the 2017 rockfall event sequence from El Capitan because following the collapse of a 180 m^3^ rock sheet, another 23-m-high and 14-m-wide rock sheet (10-cm-thick) located immediately above the rockfall scar was also affected by a rotational movement along the vertical axis^79^. Thus, this type of tearing mechanism that occurs at the failure time not only affects thin rock slabs but can also affect rock compartments that are tens of meters thick.

### Temporal evolution of rockfall source number and eroded volume

The histograms in Fig. 5 provide better visualization of the three periods of rock failure activity that characterize the 11 years of monitoring. As already discussed above, two periods of rock failure activity decay (2005–2008, then 2011–2016; now called Period 1 and Period 3, respectively) bracket a destabilization phase in 2008–2011 (Period 2). Whether for Period 1 or Period 3, the annual evolution of the rockfall source number after a major collapse follows a rapid exponential decay (Fig. 5b): for Period 1, this number was divided by a factor of 4 within three years, and for Period 3, this number was divided by a factor of 17.5 during the same time interval (Table 2). Based on these two exponential decay laws, the theoretical decay time to reach a new state of slope stability (annual rock failure activity close to zero) is 8.2 ± 0.2 years for an event of approximately 300,000 m^3^ and 3.4 ± 0.6 years for an event of approximately 40,000 m^3^. The trend is similar for the annual evolution of the eroded volume, but this time (the values of R^2^ being higher with power-law decays than with exponential decays: 0.97 instead of 0.87 for Period 1 and 0.87 instead of 0.70 for Period 2), the decrease follows a power-law decay (Fig. 5d). Furthermore, it is interesting to highlight that the slopes of both fitted power-law relationships for Period 1 and Period 3 are almost identical (*β*_*Ev*_ = 6.42 and 6.23) and that the annual eroded volume decreased by four to five orders of magnitude within three years.

Due to the progressive search for a new slope stability imposed by a major event^45,95^, this kind of decay (for both the number of post-failure events and the eroded volume) may seem to be a fairly natural trend: small-scale mechanical readjustments would thus take place quickly after a significant destabilization. However, it should be specified that this phenomenon has been little observed for rockfall scars and even less quantified. A relevant example^92^ comes from Yosemite Valley. Following a major rockfall event of more than^40^ 20,000 m^3^, which affected the southeast face of Middle Brother in March 1987, the U.S. National Park Service monitored (daily observations) the post-failure rockfall activity for 3 months. According to this survey, the overall decay in the average rate of daily rockfall event number followed a negative power-law relationship with a *β*_*N*_ value of 1.58 (R^2^ = 0.62). In our study, power-law fitting curves were also tested for the annual number of rock failures, but for both Period 1 and Period 3, the values of R^2^ were higher with exponential decays (0.98 instead of 0.91 for Period 1; 0.77 instead of 0.40 for Period 2). In any case, the decay in rock failure activity after a large rockfall event reflects the redistribution of stresses within the rock mass, which tends to reach a new state of stability. Well-known phenomenon in the field of seismology, this mechanism of stress redistribution is regularly observed after a main shock with a decrease in aftershock activity^96–98^.

However, the high number of rockfall sources detected between 2005 and 2016 should not obscure the fact that our monitoring is subject to several biases. The first is related to the differences between the two viewpoints because even though there are some overlapping areas between the FP and DG point clouds, the two topographies complement each other. Thus, the areas masked by the lateral perspective of the 2005–2011 period acquisitions are not the same as those of the 2011–2016 period (frontal perspective). Furthermore, before November 2011, the entire lower part of the rock wall was not scanned (Fig. 2b), which means that the rock failures that affected the base of the rock face between 2005 and 2011 were not detected. By contrast, due to the shorter distance from the rock face, the resolution of point clouds acquired before November 2011 is 1.4 times higher. This factor, along with a better LoD_95%_ (± 2.7 cm instead of ± 3.5 cm), explains why rockfall sources below 0.01 m^3^ were only detected between October 2005 and September 2011 (Table 2). Due to this difference in resolution, it is therefore likely that the number of small rock failures involved during the 2011–2016 period is strongly underestimated.

The other sources of bias are due to the effects of coalescence and superimposition^42^ because our infrequent interval of monitoring (one acquisition per year) likely exceeds the return periods of many rock failures. Thus, it is likely that a large number of rock failures (and the probability increases with larger volumes) detected as single events have actually detached into several pieces (aggregated and/or superimposed). The two aforementioned effects tend to decrease the total number of rock failures detected and to increase the volume of certain events. Due to the technical problem in September 2009, the 2008–2010 period (Fig. 3) is therefore more subject to the effects of coalescence and superimposition than other periods, especially the 2228 m^3^ volume that could be linked to more than one event even though the main event was precisely dated. For the major collapses in September and October 2011, these effects are proven since the network of rockfall observers in the Mont Blanc massif reports three rockfall events on 10–11 September 2011 and two rockfall events on 29–30 October 2011. However, despite this information, no photograph allowed to precisely delimit each event. Last, it is important to again specify that the coalescence and superimposition effects do not influence the values of cumulative rock failure volumes (i.e., those shown in Table 2 and Fig. 5). Thus, this difference with the number of detected rock failures emphasizes the high reliability of the results presented in Fig. 5.

### Rockfall source volume-frequency distributions

**Figure 8:**
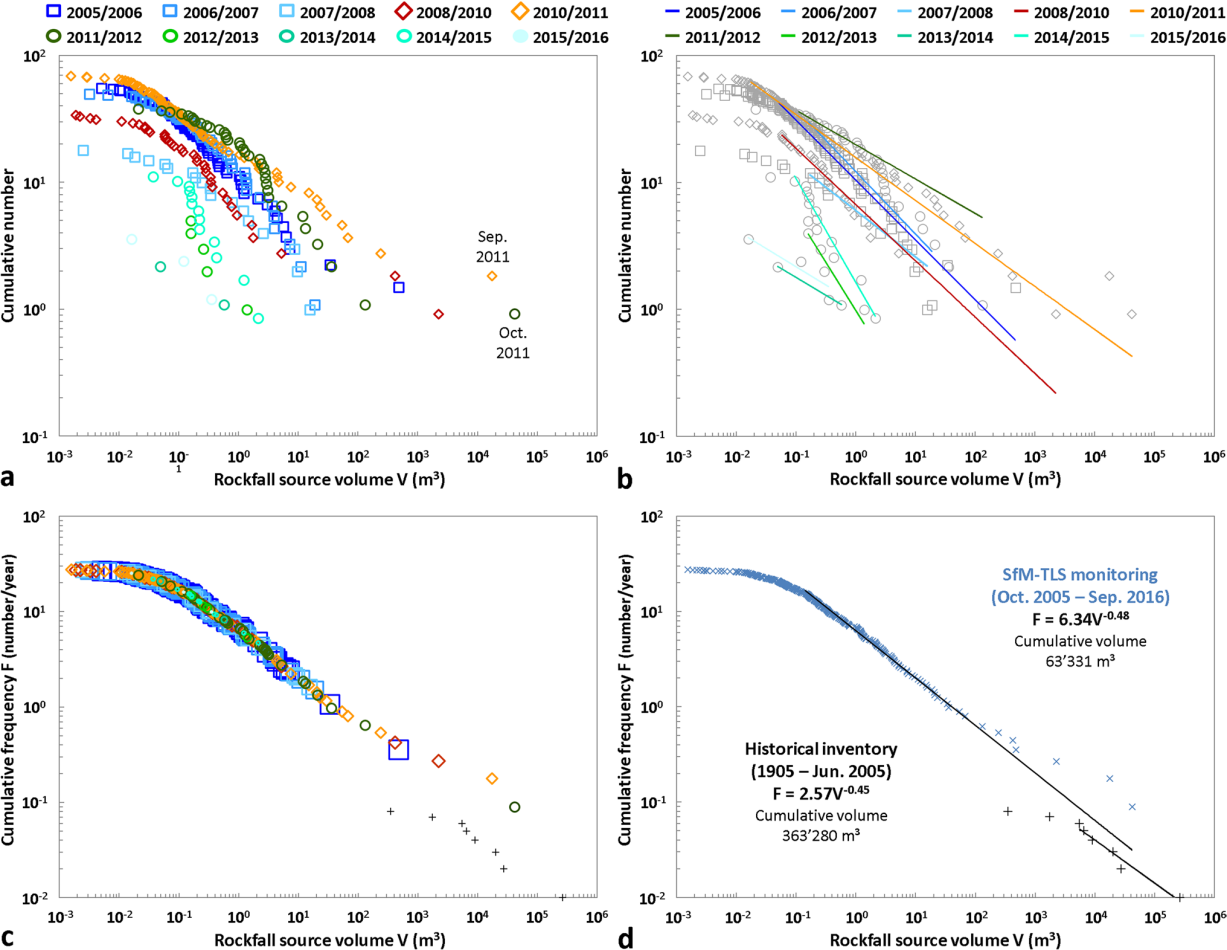
Rockfall^20,47^ source volume-frequency relationships for the Drus west face between Jun. 2005 and Sep. 2016. (**a**), (**b**) Cumulative distribution for each monitored period fitted with power laws (Panel (**b**)) using the maximum likelihood method. Power-law parameters (*α*, *β*, R^2^) are shown in Fig. 9b; their domains of validity and goodness-of-fit indicators are specified in Supplementary Table 2. (**c**), (**d**) Cumulative distributions for the time periods of 1905–Jun. 2005 and Oct. 2005-Sep. 2016 fitted with power laws (Panel (**d**)) using the maximum likelihood method. Black data points are from the historical inventory (Fig. 1c). Goodness-of-fit indicators: 1905–2005: R^2^ = 0.971, SSE = 0.087, RMSE = 0.147; 2005–2016: R^2^ = 0.997, SSE = 0.267, RMSE = 0.040.

**Figure 9:**
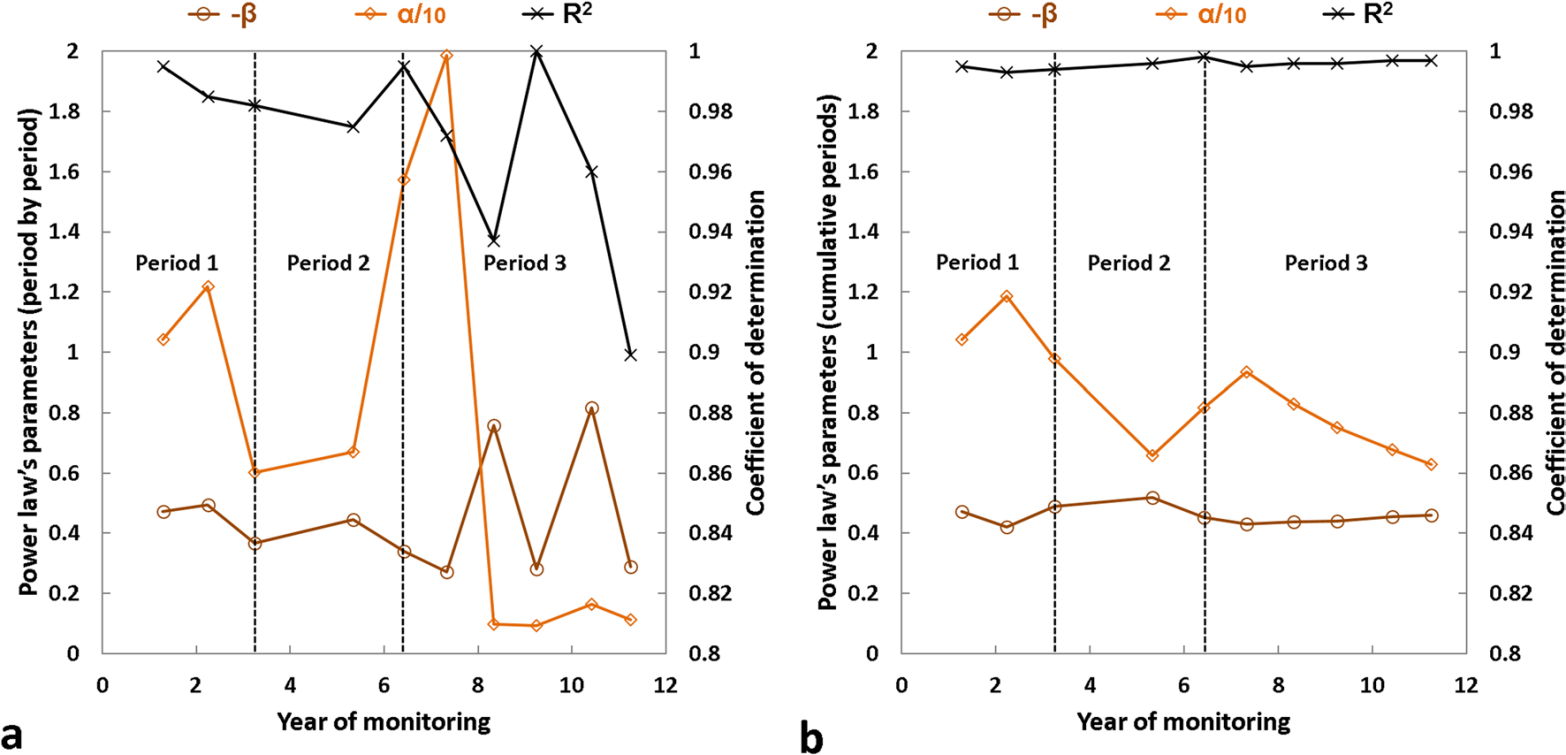
Rockfall source power law statistics determined between 2005 and 2016 in the Drus west face. Temporal evolution of parameters *α* and *β* and the coefficient of determination R^2^ by considering each monitored period as independent (**a**) or by cumulating the periods (**b**). The values of *α* and *β* are on the left y-axis, and the values of R^2^ are on the right y-axis; *α*-values have been divided by ten to appear on the graphs. The domains of validity and goodness-of-fit indicators of each power law are specified in Supplementary Tables 1 and 2, respectively.

The analysis of rockfall source frequencies was carried out based on the following two cases: (1) each monitored period is considered to be independent, and (2) all monitored periods are cumulative. Directly influenced by the temporal evolution of the rock failure number during monitoring, the results of the first analysis (Figs. 8 and 9; see also Supplementary Table 2) allow the three periods described above to be dissociated. Thus, although the goodness-of-fit indicators are low (R^2^ < 0.970; RMSE > 0.150; see also Supplementary Table 2) or biased (R^2^ = 1 in 2013–2014 because only two sources were detected) for the last four years of monitoring (which was expected given the small numbers of rock failures detected between 2012 and 2016), the *α*-value decreases (overall) during Period 1 and Period 3 and increases during Period 2 (Figs. 8a and 9a). However, Fig. 9a very clearly shows that the curve representing the variations in the *α*-value is shifted in time by one year compared with the three defined periods. Therefore, although the rock failure number is lower in 2006–2007 than in 2005–2006 (and the same for 2011–2012 versus 2010–2011), the *α*-value continues to increase. In both cases, this trend is due to a rise in the proportion of small rock failures compared with larger rock failures (Fig. 8a). This result indicates that despite the large volumes that collapsed in June 2005 and September–October 2011, the majority of the post-collapse activity consisted of small rock failures whose volumes were less than 1 m^3^. Regarding variations in the *β*-value, no clear correlation could be established with those of the *α*-value (Fig. 9a) for the first analysis. Nevertheless, it is interesting to note that the *β*-value does not vary greatly and oscillates only between 0.47 and 0.27 from 2005 to 2012 (Fig. 8b; see also Supplementary Table 2). Beyond 2012, the low goodness-of-fit indicators do not allow for further interpretation (Fig. 9a).

**Figure 10:**
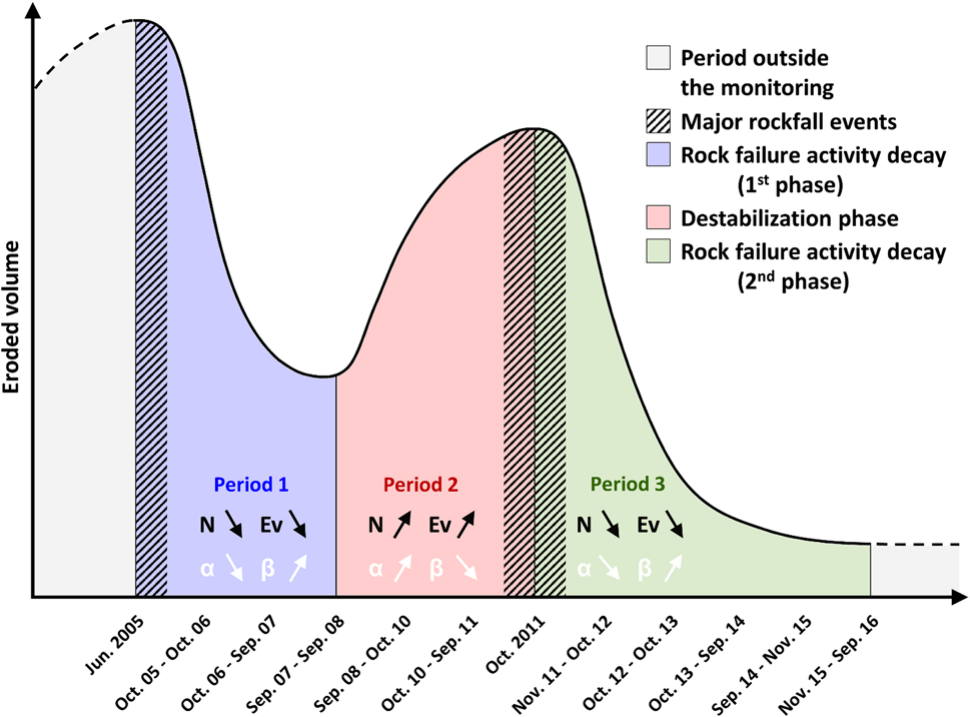
Conceptual model of the erosion process that affected the Drus west face between 2005 and 2016. The eroded volume is represented by a logarithmic scale. Ev = annual eroded volume; N = annual number of rockfall sources; α = *α*-value; β = *β*-value. The down and up arrows indicate a decrease and an increase, respectively. A simultaneous decrease in N and Ev values marks a phase of rock failure activity decay; a simultaneous increase in N and Ev values marks a destabilization phase. During a phase of rock failure activity decay, the *α*-value gradually decreases since the *N*-value decreases with time. The progressive increase in the *β*-value during such a period indicates a rise in the proportion of small volumes compared with larger volumes, and thus a progressive decrease in the *Ev*-value; for the Drus west face, the majority of the post-collapse activity consisted of small rock failures whose volumes were less than 1 m^3^. During a destabilization phase, the *α*-value gradually increases since the *N*-value increases with time. The decrease in the *β*-value during such a period indicates a gradual rise of rockfall source volumes, which results in an increase in the *Ev*-value.

The results of the second analysis (Fig. 9b; see also Supplementary Table 3) can also be divided according to the three defined periods. Furthermore, in a similar way (but much clearer) to the results of the first analysis, the *α*-value decreases (with a one-year time lag) during Period 1 and Period 3 and increases (with a one-year time lag) during Period 2 (Fig. 9b). However, unlike the first analysis, the temporal evolution of the *α*-value is anti-correlated with that of the *β*-value. Thus, although all the *β*-values are within a limited range ([0.42; 0.52]; see Supplementary Table 3), the *β*-value decreases when the *α*-value increases, and vice versa. The one-year time lag observed for the cumulative periods is probably the consequence of the effects of coalescence and superimposition effects because by distorting the number of detected rock failures and their individual volumes, they directly influence the parameters of power laws^42^. Statistically, the monitoring years most prone to these effects are those with the largest number of rock failures and/or the highest volumes since the less rock failures there are, the greater the probability that the determined volumes correspond well with individual events. In our study, these periods are the first year of monitoring and the three years characterizing the destabilization phase. Assuming that all the largest volumes detected during these four years of surveying have in fact collapsed into multiple pieces, the *α*-values would significantly increase and would likely remove the observed shift. The redistribution of volumes within the power law (which should contain more volumes larger than 1 m^3^) would simultaneously modify the *β*-values (which should therefore decrease). Consequently, the simultaneous evolution of parameters *α* and *β* observed in this study can be summarized as follows: (1) during a phase of rock failure activity decay, the *α*-value progressively decreases and the *β*-value increases; and (2) during a destabilization phase, the *α*-value increases and the *β*-value decreases. This simple conceptual model that characterizes the erosion process that affected the Drus west face between 2005 and 2016 is synthesized in Fig. 10. Naturally, this trend needs to be confirmed with high frequency surveys such as those implemented for railways^41^ and coastal cliffs^42^; however, for technical reasons, this kind of monitoring is difficult to implement at high altitude.

The TLS-derived rock failure frequency obtained for the Drus west face over the 2005–2016 period is very well fitted (166 events; R^2^ = 0.997; RMSE = 0.040; see Supplementary Table 3) by a power-law relationship between 0.1 and 100 m^3^ (Fig. 8d). Nevertheless, it should be specified that the distribution of volumes is wavy within the fitting range since the value of the SSE indicator is greater than 0.2 (see Supplementary Table 3). As with many studies^82,99–101^, the distribution is characterized by a flattening of the curve (commonly known as “rollover”) for small volumes (here < 0.1 m^3^). The rollover is due to a censoring effect^102^ and reflects an undersampling attributable to observation biases, part of which includes the monitoring frequency. Over the 2005–2016 period, the power-law *β*-value is 0.48, and its *α*-value (which reflects the average rock failure activity when considering V = 1 m^3^ in Eq. (1)) is 6.3 rock failures larger than 1 m^3^ per year (Fig. 8d). These values (especially the *β*-value) are very close to those obtained for the historical rockfall events over the 1905–2005 period^20^, namely, a *β*-value of 0.45 and an *α*-value of 2.6 rock failures larger than 1 m^3^ per year (Fig. 9b). By way of comparison with the Cretaceous granitic cliffs of Yosemite National Park, the power law associated with the 1915–1992 period (catalog of 101 events^31^) is characterized by a *β*-value of 0.46 and an *α*-value of 4.5 rock failures larger than 1 m^3^ per year. In the case of the southeast face of El Capitan mentioned above, one recent study^40^ has determined by means of SfM-TLS monitoring a power law characterized by a *β*-value of 0.41 and an *α*-value of 1.2 rock failures larger than 1 m^3^ per year over the 1976–2017 period. Although significant differences exist between these four databases (number of rockfall sources, volume range, length of the observation period, average elevation of rock faces—the highest peak in Yosemite Valley is just over 3000 m a.s.l., failure mechanisms, and age of the granites), the comparison between these rock failure frequencies seems to show similarities between the erosion processes that shape the granitic rock faces of medium and high mountains.

### Cliff retreat rates

**Figure 11:**
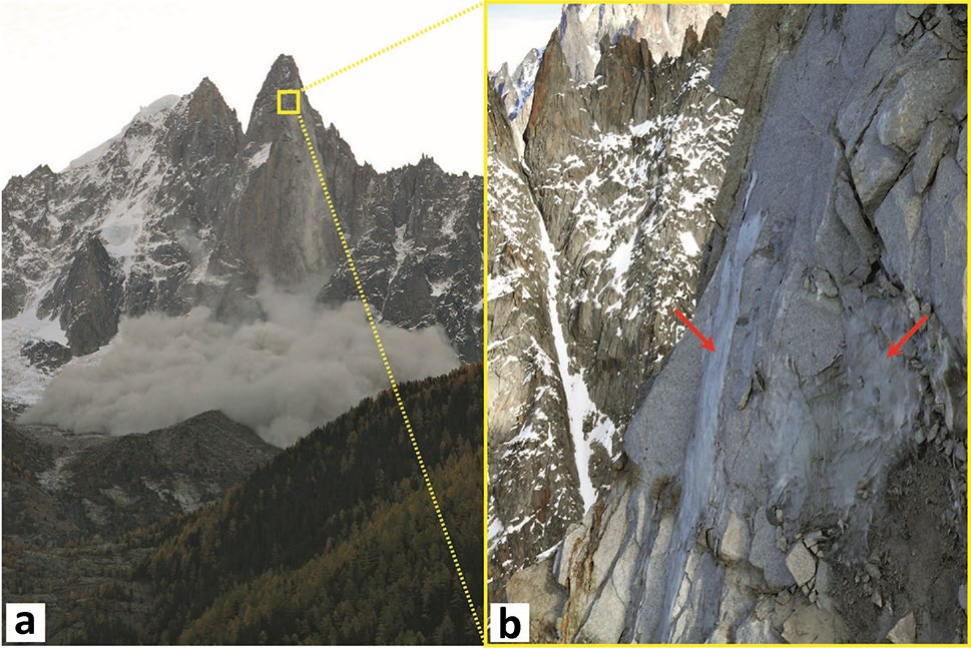
The rockfall event of 30 Oct. 2011 (41,810 m^3^). (**a**) The cloud of dust clearly visible from the Chamonix-Mont-Blanc valley; photographic credit: Sylvain Chapeland (photograph reproduced under an open access license CC BY). (**b**) A massive amount of ice (red arrows) was present at the top of the scar, indicating permafrost conditions.

The cumulative eroded volume measured between October 2005 and September 2016 is 63,331 m^3^ (Fig. 8d; Table 2). Between 1905 and June 2005, the historical inventory gives a cumulative volume of 363,280 m^3^ (Figs. 1c and 8d). Thus, in the Drus west face, a total volume of 426,611 m^3^ collapsed between 1905 and 2016. By considering the surface area measured by TLS from the DG viewpoint, this last value corresponds to a rock wall retreat rate of 14.4 mm year^−1^. Between June 2005 and October 2011, the retreat rate is nearly nine times faster since its value is 121.3 mm year^−1^. Deeply influenced by the volume of the June 2005 rockfall event, these rock wall retreat rates are very high compared to those^33,82,103–107^ usually measured in other mountainous regions, which typically vary from 0.01 to < 1.5 mm year^−1^. Comparing the values is complicated because the methods used to obtain these results are different, while the representativeness of the measures on which the calculations are based may be insufficient (small study areas, seasonal measures) compared with the phenomena studied^108^. Nevertheless, the very fast retreat rate of the Drus west face probably results from accelerated Alpine permafrost degradation^109–111^ since the early 1990s with ongoing climate change^112^, which makes thermocryogenic processes prepare and trigger rock failures^17,18,28,113^. The west face of the Drus is indeed permafrost-affected, as shown by a statistical model^114^ and temperature measurements carried out 20 m downstream from the scanner position (see Fig. 2b), on the north-west slope of the Flammes de Pierre ridge. Between 15 October 2006 and 13 October 2009, the average temperature of the rock at a depth of 55 cm was − 2.8 °C, indicating conditions of cold permafrost but corresponding to a temperature shown as very favorable to the rockfall triggering^28^. On the one hand, one study^20^ reported that no ice indicating permafrost conditions was directly observed in the scar for the June 2005 rockfall event, but water seepage persisted throughout the summer along the scar without heavy rainfalls, suggesting melting of the ice that was previously present in the fractures before the collapse. Moreover, there was a strong correlation between the pre-2005 rock failure occurrences and the warmest periods over the last 100 years. A paraglacial control affecting the rock wall^45,115–117^ is to be excluded since the Last Glacial Maximum trimline is located 300 m below the 1950 rockfall scar. The same is true for seismicity. According to the *SisFrance* seismic monitoring network, 23 earthquakes with an intensity greater than or equal to III were recorded in Chamonix during the 1850–2005 period but none directly triggered a rockfall^20^. In addition, over the 2005–2016 period, no precisely dated event matches an earthquake recorded by the *SISMalp* and *RéNaSS* networks for the Mont Blanc region. While the role of earthquakes in preparing for collapse is probable, it is difficult to measure it. Thus, permafrost degradation caused by the present climatic warming is probably the main triggering factor of most of the main Drus rock failures. For 2005, permafrost degradation was more frequent and deep-seated because the Bonatti Pillar received a strong heat flux on its southern aspect, and the densely fractured granite promoted active water drainage and heat transfer by advection into the rock mass^17,118^. On the other hand, the 2005 event released a very large volume, which allowed for the establishment of a new active layer (surface layer of permafrost that thawed each summer). Thus, the deepening of this active layer^28^ could take part in triggering certain events, such as those 2011, since ice was observed within the 30 October 2011 rockfall scar (Fig. 11). Note that the structural arrangement of the rock face favors the formation of subvertical overhanging rock pillars^47,54^ prone to instabilities.

Different time scales of observation can affect the relevance of the rock wall retreat rate comparison from various rock failure inventories. To assess a long-term averaged total eroded volume, we followed the method^36^ of integrating the frequency density multiplied by the rock failure volume *V*. By considering a time window of 1000 years with a minimum volume of 10^−3^ m^3^ and a maximum volume of 10^6^ m^3^, a long-term averaged total volume of 771,500 m^3^ was determined. This eroded volume corresponds to a long-term retreat rate of 2.9 mm year^−1^, which is a value clearly closer to the range of retreat rates discussed above. In agreement with several studies^36,40,82^, the long-term averaged volume-frequency relationships derived from remote sensing surveys seem to be an accurate way to quantify rock failure erosion within mountainous landscapes.

## Conclusions

The implementation of TLS monitoring over an 11-year period has made it possible to precisely quantify the spatial and temporal evolution of the rock failure activity that affected the Drus west face following the large rockfall event of June 2005. By comparing high-resolution terrain models year after year, 307 rockfall sources ranging from 0.002 to 41,810 m^3^ were detected between October 2005 and September 2016. This time window is divided into the following three periods: a phase of rock failure activity decay until September 2008, a destabilization phase between September 2008 and November 2011, and a new phase of rock failure activity decay from November 2011 to September 2016. The destabilization phase led to the collapse of a pillar of 61,494 m^3^ located in the northern part of the June 2005 rockfall scar. Similar to the progressive collapse of the Bonatti Pillar, in which the June 2005 event is highlighted, rock failure instability propagated upward (retrogressive erosion) with increasing volumes. The two phases of rock failure activity decay are characterized by a number of rock failures that decrease exponentially and by an eroded volume that decreases following a power-law distribution. A power law fitted over 166 events describes the distribution of volumes detected between 2005 and 2016 with an exponent of 0.48 and an average rock failure activity of more than six events larger than 1 m^3^ per year. Following the intense rock failure activity that has affected the rock face since the beginning of the twentieth century, the determined rock wall retreat rate is much faster than the retreat rates measured in other high mountainous regions. Thus, even though the rock failure activity of the Drus west face is distinguished by its exceptional nature, the TLS monitoring performed made it possible to characterize the progressive research of a new slope stability imposed by a major rockfall event of almost 300,000 m^3^ with an unprecedented level of detail. Although subject to detection bias due to its low frequency, our long-term monitoring has enabled us to collect valuable information on rock failure frequencies within high altitude granitic rock faces, thereby contributing to improving our understanding of landscape evolution in mountainous regions.

## Supplementary information

Supplementary Information.

## Data Availability

The datasets generated during and/or analyzed during the current study are available from the corresponding author on reasonable request.
