# Supplementary material for: The three-stage rock failure dynamics of the Drus (Mont Blanc massif, France) since the June 2005 large event
Source: Sci Rep. 2020 Oct 15;10:17330. doi: 10.1038/s41598-020-74162-1 (PMC7567073; doi:10.1038/s41598-020-74162-1)
Supplement: Supplementary file 1 — Supplementary Information. [file 41598_2020_74162_MOESM1_ESM.docx]

**The three-stage rock failure dynamics of the Drus (Mont Blanc massif, France) since the June 2005 large event**

**Antoine Guerin, Ludovic Ravanel, Battista Matasci, Michel Jaboyedoff & Philip Deline**

**Supplementary information**

**Supplementary Table 1:** Representative sample of rockfall source volumes and volumetric errors for the 2005-2006 period.

**Supplementary Table 2:** Characteristics of rockfall source volume-frequency distributions (independent periods).

**Supplementary Table 3:** Characteristics of rockfall source volume-frequency distributions (cumulative periods).

**Supplementary Figure 1:** Technical features of the Oct. 2005 TLS point cloud.

**Supplementary Figure 2:** Technical features of the autumn 2011 TLS point clouds.

**Supplementary Figure 3:** Rockfall volume calculation method.

**Supplementary Table 1. Representative sample of rockfall source volumes and volumetric errors for the 2005-2006 period.** C = cubic; Cx = complex; RP = rectangular parallelepiped; TP = triangular prism. The dimensions specified in the last three columns correspond to those of the approximate geometric shape.

| **Volume**  **(m³)** | **Volumetric error**  ± **(m³)** ± **(%)** | | **Approximate shape** | **Length**  **(m)** | **Width**  **(m)** | **Depth**  **(m)** |
| --- | --- | --- | --- | --- | --- | --- |
| 476 | 8.1 | 1.7 | RP | 30 | 10 | 1.59 |
| 34 | 1.1 | 2.9 | Cx = C + RP | 4.0 + 2.0 | 4.0 + 10 | 0.60 + 1.22 |
| 7.1 | 0.22 | 3.1 | RP | 4.0 | 2.0 | 0.89 |
| 6.2 | 0.27 | 4.3 | TP | 5.5 | 3.5 | 0.65 |
| 5.6 | 0.20 | 3.6 | RP | 3.8 | 2.1 | 0.70 |
| 4.4 | 0.17 | 3.9 | Cx = C + RP | 2.0 + 2.5 | 2.0 + 1.0 | 0.78 + 0.50 |
| 4.1 | 0.22 | 5.3 | TP | 4.0 | 2.0 | 0.78 |
| 3.0 | 0.11 | 3.7 | C | 2.0 | 2.0 | 0.75 |
| 2.1 | 0.13 | 6.2 | RP | 5.0 | 1.0 | 0.42 |
| 1.2 | 0.06 | 4.9 | RP | 2.2 | 0.9 | 0.62 |
| 1.2 | 0.08 | 6.7 | RP | 3.0 | 1.0 | 0.40 |
| 1.1 | 0.06 | 5.4 | C | 1.6 | 1.6 | 0.43 |
| 1.1 | 0.06 | 5.5 | RP | 1.9 | 1.2 | 0.50 |
| 1.0 | 0.05 | 5.4 | RP | 2.8 | 0.8 | 0.50 |
| 0.5 | 0.04 | 8.5 | RP | 2.3 | 0.6 | 0.34 |
| 0.1 | 0.01 | 10.9 | RP | 1.3 | 0.3 | 0.26 |
| 0.05 | 0.008 | 15.7 | RP | 1.0 | 0.3 | 0.17 |
| 0.01 | 0.002 | 20.1 | RP | 0.3 | 0.2 | 0.16 |
| 0.005 | 0.001 | 27.4 | RP | 0.3 | 0.2 | 0.10 |

**Supplementary Table 2. Characteristics of rockfall source volume-frequency distributions (independent periods).** N_fit_ = number of events used for power law fitting; V_fit_ = volume range used for power law fitting; α, β = maximum likelihood estimates of *α*-value and *β*-value; SSE = sum of squared estimate of errors; RMSE = root mean square error.

| **Period** | **N_fit_** | **V_fit_ (m³)** | **α** | **β** | **R^2^** | **SSE** | **RMSE** |
| --- | --- | --- | --- | --- | --- | --- | --- |
| Oct. 05 - Oct. 06 | 52 | 0.05 – 5.7 | 10.43 ± 0.27 | 0.47 ± 0.07 | 0.995 | 0.098 | 0.044 |
| Oct. 06 - Sep. 07 | 24 | 0.16 – 3.9 | 12.18 ± 0.46 | 0.49 ± 0.10 | 0.985 | 0.085 | 0.062 |
| Sep. 07 - Sep. 08 | 10 | 0.17 – 8.8 | 6.01 ± 0.43 | 0.37 ± 0.12 | 0.982 | 0.034 | 0.065 |
| Sep. 08 - Oct. 10 | 24 | 0.05 – 5.3 | 6.69 ± 0.62 | 0.44 ± 0.09 | 0.975 | 0.212 | 0.098 |
| Oct. 10 - Nov. 11 | 48 | 0.02 – 0.7 | 15.72 ± 0.50 | 0.34 ± 0.05 | 0.995 | 0.035 | 0.028 |
| Nov. 11 - Oct. 12 | 20 | 0.11 – 2.5 | 19.87 ± 0.63 | 0.27 ± 0.06 | 0.972 | 0.040 | 0.047 |
| Oct. 12 - Oct. 13 | 4 | 0.16 – 1.4 | 0.98 ± 0.97 | 0.76 ± 0.38 | 0.937 | 0.069 | 0.185 |
| Oct. 13 - Sep. 14 | 2 | 0.05 – 0.6 | 0.93 | 0.28 | 1 | - | - |
| Sep. 14 - Nov. 15 | 12 | 0.09 – 2.2 | 1.63 ± 0.68 | 0.82 ± 0.24 | 0.960 | 0.249 | 0.158 |
| Nov. 15 - Sep. 16 | 3 | 0.01 – 0.4 | 1.12 ± 0.78 | 0.29 ± 0.17 | 0.899 | 0.063 | 0.205 |

**Supplementary Table 3. Characteristics of rockfall source volume-frequency distributions (cumulative periods).** N_fit_ = number of events used for power law fitting; V_fit_ = volume range used for power law fitting; α, β = maximum likelihood estimates of *α*-value and *β*-value; SSE = sum of squared estimate of errors; RMSE = root mean square error.

| **Period** | **N_fit_** | **V_fit_ (m³)** | **α** | **β** | **R^2^** | **SSE** | **RMSE** |
| --- | --- | --- | --- | --- | --- | --- | --- |
| Oct. 05 - Oct. 06 | 52 | 0.05 – 5.7 | 10.43 ± 0.27 | 0.47 ± 0.07 | 0.995 | 0.098 | 0.044 |
| Oct. 05 - Sep. 07 | 72 | 0.05 – 1.3 | 11.86 ± 0.26 | 0.42 ± 0.05 | 0.993 | 0.085 | 0.035 |
| Oct. 05 - Sep. 08 | 65 | 0.15 – 6.3 | 9.79 ± 0.14 | 0.49 ± 0.06 | 0.994 | 0.112 | 0.042 |
| Oct. 05 - Oct. 10 | 82 | 0.15 – 7.2 | 6.59 ± 0.07 | 0.52 ± 0.06 | 0.996 | 0.089 | 0.033 |
| Oct. 05 - Nov. 11 | 118 | 0.15 – 67.8 | 8.16 ± 0.07 | 0.45 ± 0.04 | 0.998 | 0.131 | 0.034 |
| Oct. 05 - Oct. 12 | 147 | 0.15 – 67.8 | 9.34 ± 0.06 | 0.43 ± 0.04 | 0.995 | 0.350 | 0.049 |
| Oct. 05 - Oct. 13 | 153 | 0.14 – 67.8 | 8.29 ± 0.06 | 0.44 ± 0.04 | 0.996 | 0.324 | 0.046 |
| Oct. 05 - Sep. 14 | 153 | 0.14 – 67.8 | 7.50 ± 0.05 | 0.44 ± 0.04 | 0.996 | 0.301 | 0.045 |
| Oct. 05 - Nov. 15 | 165 | 0.14 – 67.8 | 6.79 ± 0.05 | 0.46 ± 0.04 | 0.997 | 0.283 | 0.042 |
| Oct. 05 - Sep. 16 | 166 | 0.14 – 67.8 | 6.29 ± 0.04 | 0.48 ± 0.03 | 0.997 | 0.267 | 0.040 |


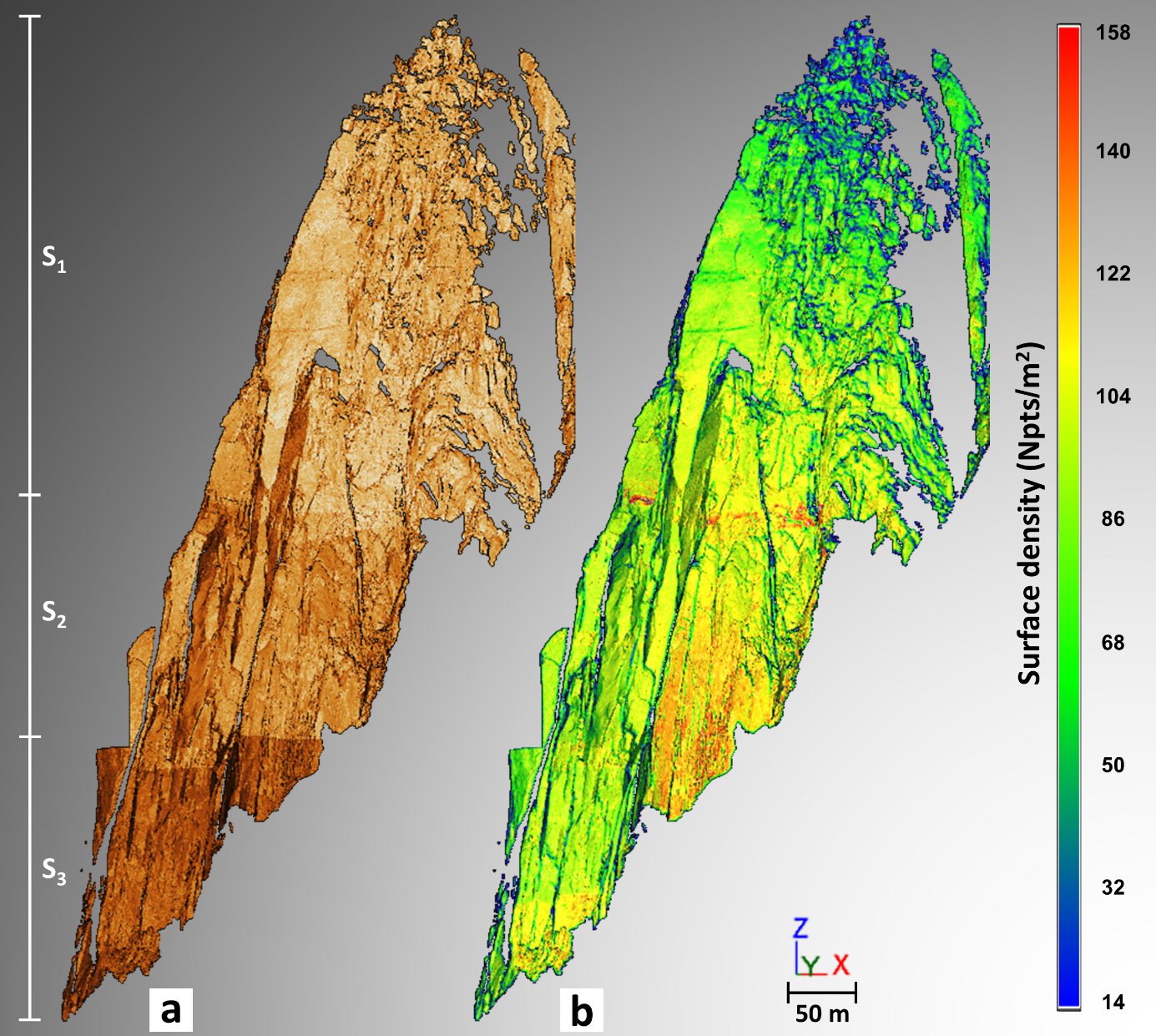


**Supplementary Figure 1. Technical features of the Oct. 2005 TLS point cloud.** **(a)** Composite point cloud (assembly of three scenes) of the Drus west face seen from the FP viewpoint (Fig. 2). Gaps in the point cloud were caused by ground areas unseen (areas masked by the relief) to the laser scanner owing to view angle. The three scenes S_1_ through S_3_ were acquired from the same position; only the laser scanner was rotated vertically. A vertical overlap of about 30% between each scene allowed them to be aligned correctly. Once the alignment procedure completed, most of the overlapping points were removed in order to obtain a more homogeneous point density (Panel *b*). **(b)** Surface density (number of points per m^2^) of the point cloud (11.3 million points) shown in Panel *a*. The average surface density (light green color) is 97 pts/m^2^. The horizontal reddish-orange band (155 pts/m^2^) visible in the center of the point cloud reflects a denser surface area due to overlapping points not removed.


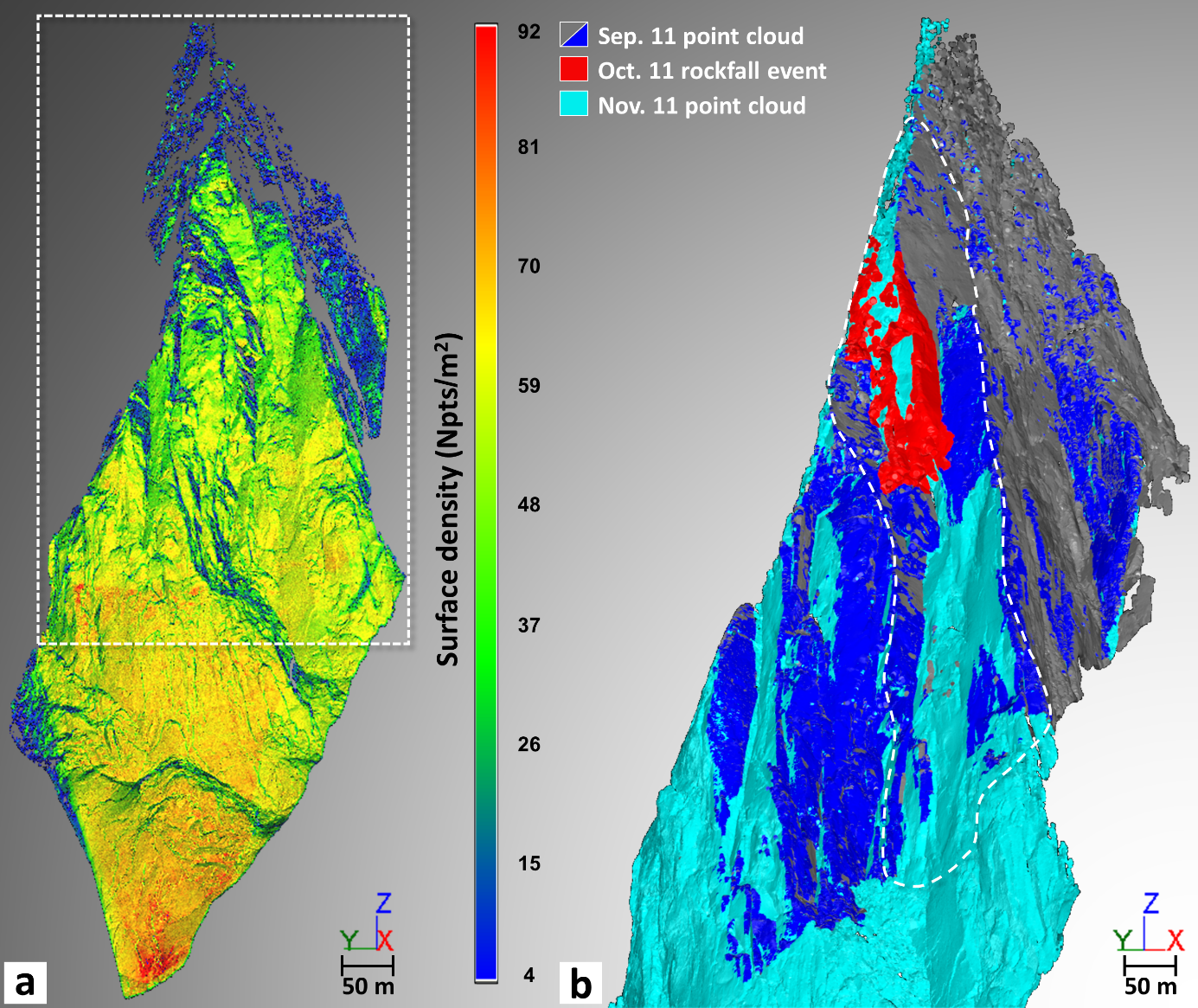


**Supplementary Figure 2. Technical features of the autumn 2011 TLS point clouds.** **(a)** Surface density (number of points per m^2^) of the Nov. 2011 point cloud (17.8 million points). The average surface density (light green color) is 61 pts/m^2^. The dashed white frame indicates the boundaries of the light blue point cloud shown in Panel *b*. **(b)** Overlapping surface area (dark blue color) between the point clouds of Sep. 2011 (FP viewpoint) and Nov. 2011 (DG viewpoint). The dashed white contour shows the boundaries of the June 2005 rockfall scar; all the dark blue points located outside this contour have been selected (stable areas) to align the Sep. 2011 and Nov. 2011 points clouds. The comparison of these two point clouds enabled the volume calculation of the 30 Oct. 2011 rockfall event. Gaps visible within the red point cloud have been filled with large triangles constrained by the radius of curvature of each hole (see Supplementary Fig. 3).


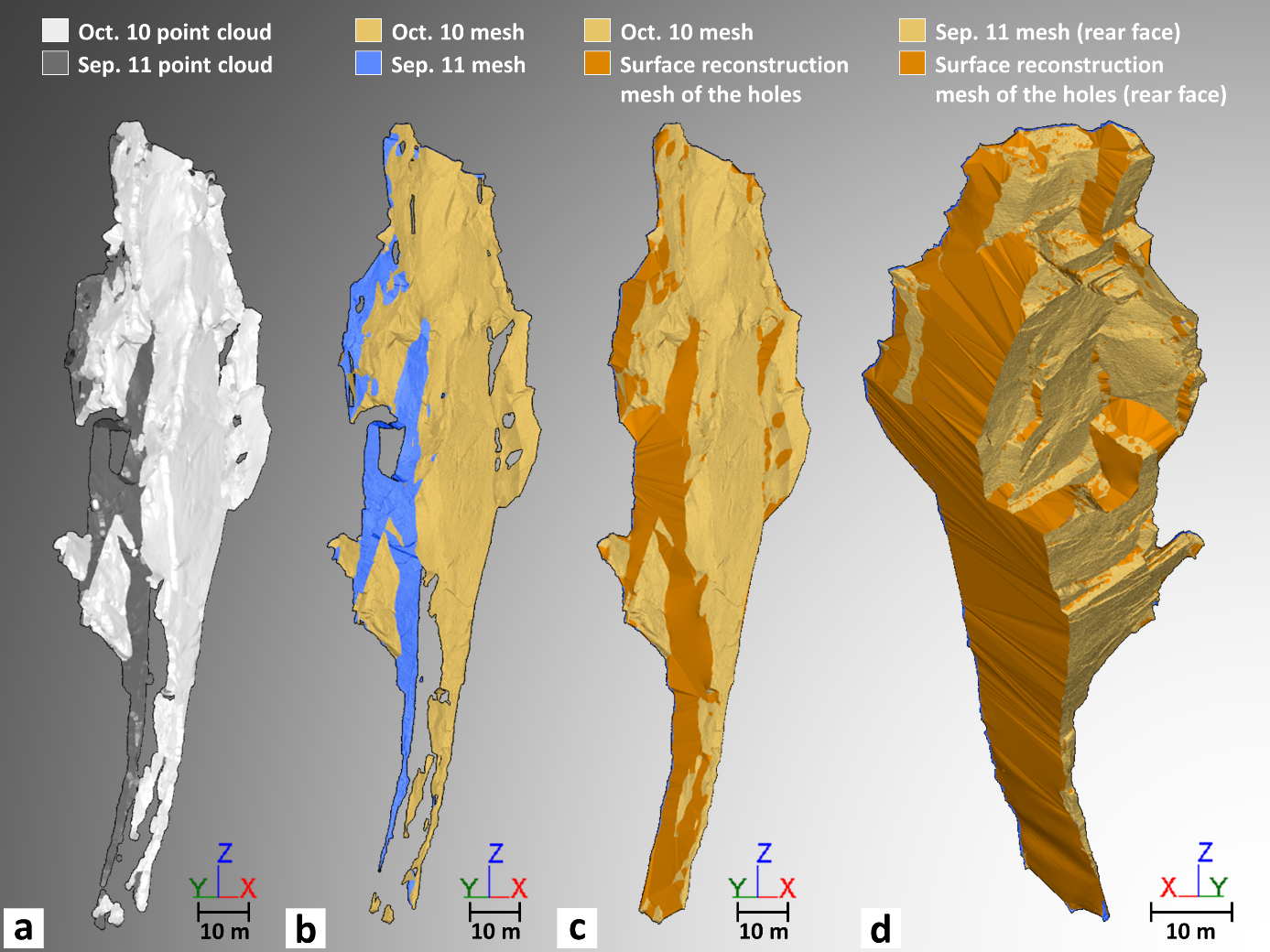


**Supplementary Figure 3. Rockfall volume calculation method.** **(a)** Side view of the collapsed surface (white color) and the scar (grey color) of the 11 Sep. 2011 rockfall event, detected from the FP viewpoint. **(b)** Triangular meshes of the two points clouds shown in Panel *a*. All the points have been kept for the generation of the meshes and at this stage, the gaps visible within the meshes have not yet been filled. **(c)** After selecting the outer contour of the two meshes, as well as those of each hole present within them (Panel *b*), a third triangular mesh (orange color) was generated with large triangles constrained by the radii of curvature fitting at best the orientation of the facets located at the edge of each hole. **(d)** Details of the side rear view of the volume of the 11 Sep. 2011 rockfall event (17,456 m³). After merging the three triangular meshes, the volume is calculated as the sum of all the tetrahedron volumes contained inside the merged mesh.
